# Metabolome and transcriptome profiling reveals anthocyanin contents and anthocyanin-related genes of chimeric leaves in *Ananas comosus* var. *bracteatus*

**DOI:** 10.1186/s12864-021-07642-x

**Published:** 2021-05-07

**Authors:** Xuzixin Zhou, Yanbin Xue, Meiqin Mao, Yehua He, Mark Owusu Adjei, Wei Yang, Hao Hu, Jiawen Liu, Lijun Feng, Huiling Zhang, Jiaheng Luo, Xi Li, Lingxia Sun, Zhuo Huang, Jun Ma

**Affiliations:** 1 College of Landscape Architecture, Sichuan Agricultural University, Chengdu, China; 2 College of Horticulture, South China Agricultural University, Guangzhou, China

**Keywords:** *Ananas comosus* var. *bracteatus*, Anthocyanin, CIELAB, Metabolomics, Transcriptomics, MYB transcription factor

## Abstract

**Background:**

*Ananas comosus* var. *bracteatus* is a colorful plant used as a cut flower or landscape ornamental. The unique foliage color of this plant includes both green and red leaves and, as a trait of interest, deserves investigation. In order to explore the pigments behind the red section of the chimeric leaves, the green and red parts of chimeric leaves of *Ananas comosus* var. *bracteatus* were sampled and analyzed at phenotypic, cellular and molecular levels in this study.

**Results:**

The CIELAB results indicated that the a* values and L* values samples had significant differences between two parts. Freehand sections showed that anthocyanin presented limited accumulation in the green leaf tissues but obviously accumulation in the epidermal cells of red tissues. Transcriptomic and metabolomic analyses were performed by RNA-seq and LC-ESI-MS/MS. Among the 508 identified metabolites, 10 kinds of anthocyanins were detected, with 6 significantly different between the two samples. The cyanidin-3,5-O-diglucoside content that accounts for nearly 95.6% in red samples was significantly higher than green samples. RNA-Seq analyses showed that 11 out of 40 anthocyanin-related genes were differentially expressed between the green and red samples. Transcriptome and metabolome correlations were determined by nine quadrant analyses, and 9 anthocyanin-related genes, including *MYB5* and *MYB82*, were correlated with 7 anthocyanin-related metabolites in the third quadrant in which genes and metabolites showing consistent change. Particularly, the PCCs between these two MYB genes and cyanidin-3,5-O-diglucoside were above 0.95.

**Conclusion:**

Phenotypic colors are closely related to the tissue structures of different leaf parts of *Ananas comosus* var. *bracteatus*, and two MYB transcription factors might contribute to differences of anthocyanin accumulation in two parts of *Ananas comosus* var. *bracteatus* chimeric leaves. This study lay a foundation for further researches on functions of MYBs in *Ananas comosus* var. *bracteatus* and provides new insights to anthocyanin accumulation in different parts of chimeric leaves.

**Supplementary Information:**

The online version contains supplementary material available at 10.1186/s12864-021-07642-x.

## Background

Anthocyanin is a prominent pigment found in plant organs and has become an indicator of the ornamental or edible value of plants [1–3]. Several enzyme-coding structural genes of anthocyanin biosynthesis have been reported in previous studies [4], and these genes were mainly distributed in three pathways, namely, phenylpropanoid biosynthesis, flavonoid biosynthesis and anthocyanin biosynthesis [5]. At the transcriptional level, anthocyanin biosynthesis is mainly regulated by MYB, basic helix-loop-helix (bHLH) and WD40 transcription factors, which form a complex to directly bind with promoters of anthocyanin biosynthetic genes, such as *chalcone synthesis* (*CHS*), *chalcone isomerase* (*CHI*), *flavanone 3-hydroxylase* (*F3H*), *flavonoid 3′-hydroxylase* (*F3′H*), *flavonoid 3′5′-hydroxylase* (*F3′5′H*), *dihydroflavonol 4-reductase* (*DFR*), *anthocyanidin reductase* (*ANS*) and *UDP-glucose: flavonoid 3-O-glucosyltransferase* (*3GT*) [6–8]. After biosynthesis, anthocyanins are transported into vacuoles by a series of transporters, including glutathione S-transferases (GSTs), multidrug resistance-associated protein (MRP), and multidrug and toxic compound extrusion (MATE) [9–11]. According to previous reports about anthocyanin related transcription factors in *Arabidopsis thaliana*, the MYB transcription factor TRANSPARENT TESTA 2 (TT2) regulates the expression of late biosynthesis genes to pigment the seed coat by combining with factors TT8 (bHLH42) and TTG1 (WD40) [12, 13]. In petunia petal cells, anthocyanin biosynthesis is also regulated by the MYB-bHLH-WD40 complex, but some MYBs, such as AtMYB12 and VvMYBF1, regulate flavonoid biosynthesis without bHLH and WD40 cofactors [14, 15]. In higher plants, anthocyanin biosynthesis is a complicated process that is easily influenced by environmental factors, including light, temperature, moisture, pH, metal ions, and plant hormones [16–20]. However, the molecular mechanism of anthocyanin accumulation in *Ananas comosus* var. *bracteatus* leaves is still largely unknown.

*A. comosus* var. *bracteatus* is an herbaceous monocot distributed primarily in tropical regions [21]. It has high decorative and ornamental value due to its chimeric leaves, which present a regular distribution of green and red colors. Therefore, *A. comosus* var. *bracteatus* is often cultivated as a commodity for the international market [22]. In addition, carbon assimilation of *A. comosus* var. *bracteatus* occurs via the crassulacean acid metabolism (CAM) pathway, thus allowing for effective utilization of water under drought conditions [23, 24]. Therefore, this plant is mostly used as an indoor potted plant or even as an outdoor landscape plant without much need for irrigation. With the increase in market demands for new varieties of colorful plants, *A. comosus* var. *bracteatus* has successfully attracted attention from researchers [25, 26], and this research is of great significance for further revealing the mechanisms of *A. comosus* var. *bracteatus* leaf color. However, detailed information on the anthocyanins in *A. comosus* var. *bracteatus* leaf tissue has not been formally reported. Moreover, the regular and specific distribution of pigments in different parts of *A. comosus* var. *bracteatus* leaves, especially the specific distribution of anthocyanins, has not been thoroughly investigated in previous research. Therefore, integrated research on the accumulation and distribution of anthocyanin in *A. comosus* var. *bracteatus* leaves is urgently needed.

Plant coloration is often measured by two main methods: Royal Horticulture Society Color Chart (RHSCC) and the CIELAB color coordinate [27]. With the development of technology, the RHSCC has gradually been replaced by advanced colorimetric devices that can digitize the brightness and saturation of color [28]. These devices based on the CIELAB color coordinate principle can accurately measure the color value by a series of indexes. Freehand cross sectioning is the prominent method used to investigate pigments in plant tissue structures and cells, allowing researchers to directly examine how pigments influence phenotypes at the cellular level. Previous studies on the dissection of the colorful leaves of *A. comosus* var. *bracteatus* have been inadequate, although studies on the anatomies of other plants, such as *Pilea cadierei* Gagnep [29]., *Aloe* [30] and *Bruguiera parviflora* [31], can be used as a reference. Additionally, molecular profiling approaches, such as metabolomics and transcriptomics, are effective for exploring the underlying mechanisms responsible for a plant’s phenotype. In recent years, LC-ESI-MS/MS and RNA-seq techniques have been rapidly developed and become popular approaches for identifying a considerably large number of plant metabolites and providing broad coverage of transcript abundance, respectively [5, 32, 33]. Transcriptome analysis combined with metabolome analysis enables the comprehensive and detailed exploration of molecular mechanisms.

To have a better understanding of anthocyanin accumulation pattern in *A. comosus* var. *bracteatus* chimeric leaves, green and red leaf parts were selected as experimental materials to carry out phenotypic, cellular and molecular analyses by CIELAB colorimeter, freehand sectioning, LC-ESI-MS/MS and RNA-seq in this study. Comparative analysis indicated the locations of anthocyanins in the leaf tissues, types of anthocyanins and genes involved in regulating anthocyanin biosynthesis in *A. comosus* var. *bracteatus* leaves. The results obtained in this study lay the foundation for molecular breeding and optimized cultivation as well as provide a reference for further research on chimeric leaf color.

## Results

### Leaf coloration

**Fig. 1:**
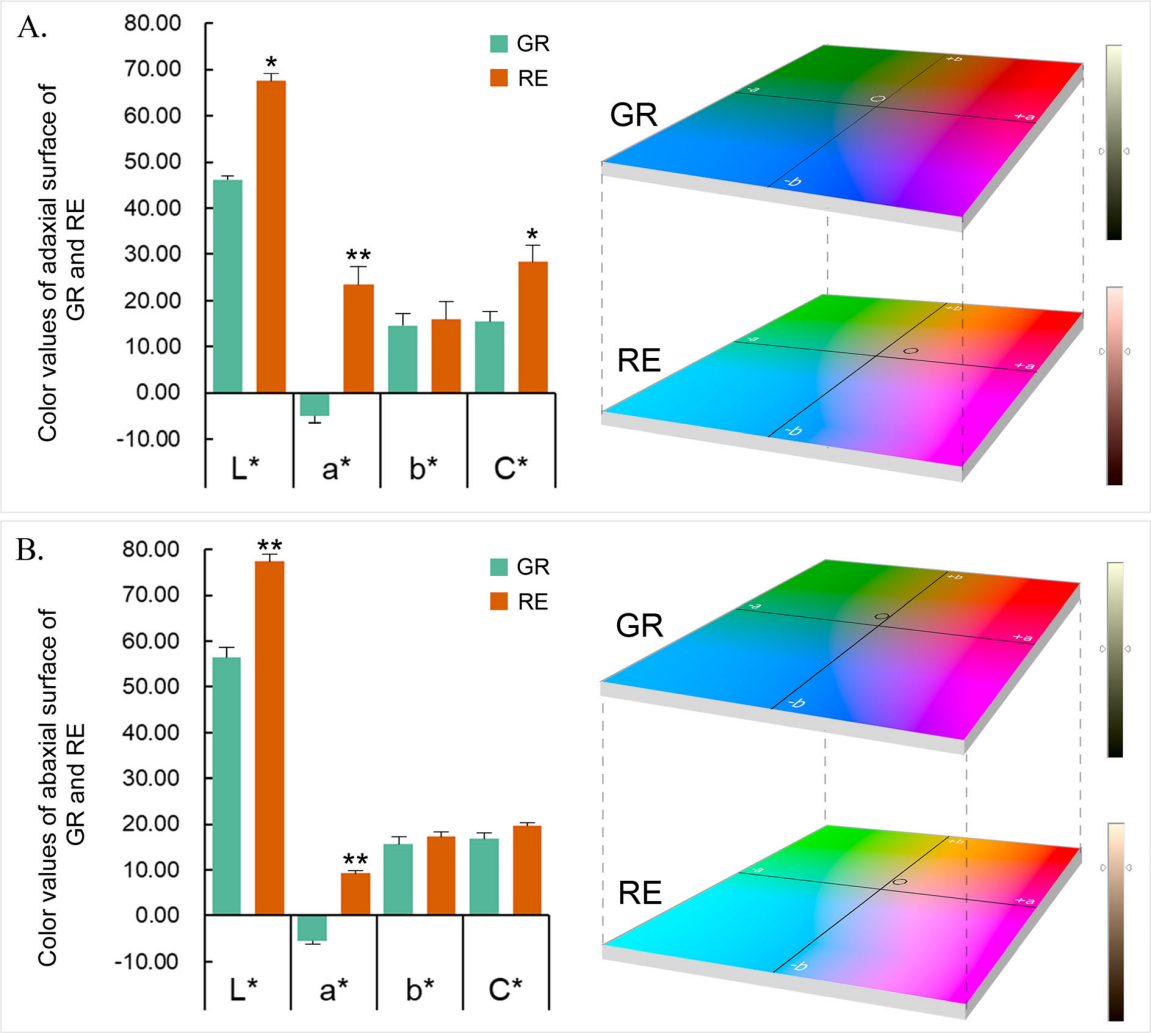
Color data of adaxial and abaxial surfaces of *Ananas comosus* var. *bracteatus* chimera leaves. **a** CIE color values and coordinate flats of the adaxial surfaces of GR and RE samples. The GR circle represents L^*^ = 46, a^*^ = −5, and b^*^ = 15; and the RE circle represents L^*^ = 68, a^*^ = 23, and b^*^ = 16. **b** CIE color values and coordinate flats of abaxial surfaces of the GR and RE samples. The GR circle represents L^*^ = 56, a^*^ = −6, and b^*^ = 16; and the RE circle represents L^*^ = 77, a^*^ = 9, and b^*^ = 17. The circle represents the rounded number of average values from triplicates of each phenotypic sample. Error bars represent ± SE of biological triplicates. Asterisks represent significant differences in gene expression between GR and RE parts (one asterisk for *P* < 0.05 and two asterisks for *P* < 0.01)

The distribution of the color values of the GR and RE samples was determined with the CIELAB coordinates: The L* values ranged from 46.03 to 77.42, the a* values ranged from − 5.68 to 23.58, the b* values ranged from 14.55 to 17.40, and the C* values ranged from 15.40 to 28.42 (Fig. 1). The L* value of GR was significantly lower than that of RE for both surfaces, indicating that the color of GR was darker than that of RE. Moreover, the a* value of GR was negative for both surfaces and that of RE was significantly higher than that of GR and was red. The b* value of both surfaces had no significant difference between GR and RE. The C* value of the RE adaxial surface was approximately 2 times higher than that of GR, but there was no significant difference between the two phenotypes on the abaxial surfaces. The CIE locations of the two samples indicated that GR mainly contained green and yellow colors, while RE mainly contained red and yellow colors.

### Anatomical cross section

**Fig. 2:**
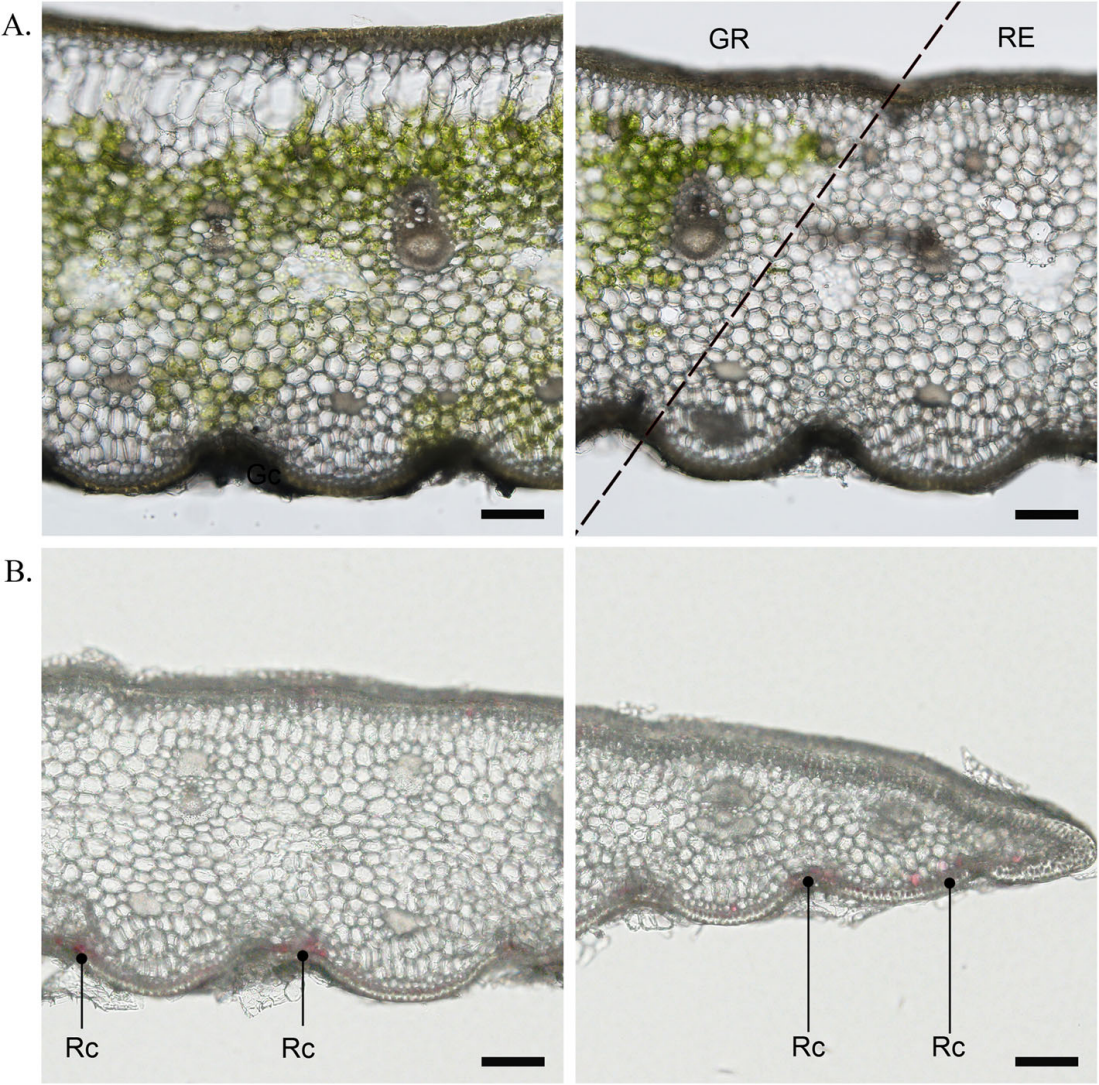
Overview of pigment distribution in chimeric leaves. **a** Cross sections of GR central and junction parts showed the chlorophyll distribution in the green mesophyll cells. The dotted line represents the boundary of the two leaf parts. **b** Cross sections of RE central and marginal parts showed red pigments (anthocyanins) located in the epidermal cells and 1–2 layers of cells under the epidermis. The bars = 100 μm. Rc, red cell

GR and RE samples were separated for anatomical analysis. The GR consisted of the epidermis, mesophyll and vein tissues (Fig. 2). In the central green part of the chimeric leaves, there were large water storage cells that are typical structural characteristics of drought-tolerant plants (Fig. 2a). Mesophyll cells of the central green part of the chimeric leaf contained chloroplasts and showed a green color. However, no water storage tissues were observed in the red margins of the chimeric leaves, and the mesophyll cells in this area did not have a green color. According to previous reports, chlorophylls and carotenoids mainly exist in photosystem I (PS I), photosystem II (PS II) and the light harvesting complex (LHC) to adjust light absorption and conversion [34–36]. These two kinds of pigments coexist together in the thylakoids, which increases the likelihood that yellow pigments, such as carotenoids, will be covered by green pigments. On the other hand, chloroplasts were invisible in freehand sections in the RE chimeric leaf samples, and the middle mesophyll mainly consisted of colorless cells (Fig. 2b). Red cells were obviously observed in the epidermis and 1–2 layers of mesophyll under the epidermis in the RE.

### Overall comparison of metabolites betwwen GR and RE samples of *A. comosus* var. *bracteatus*

**Fig. 3:**
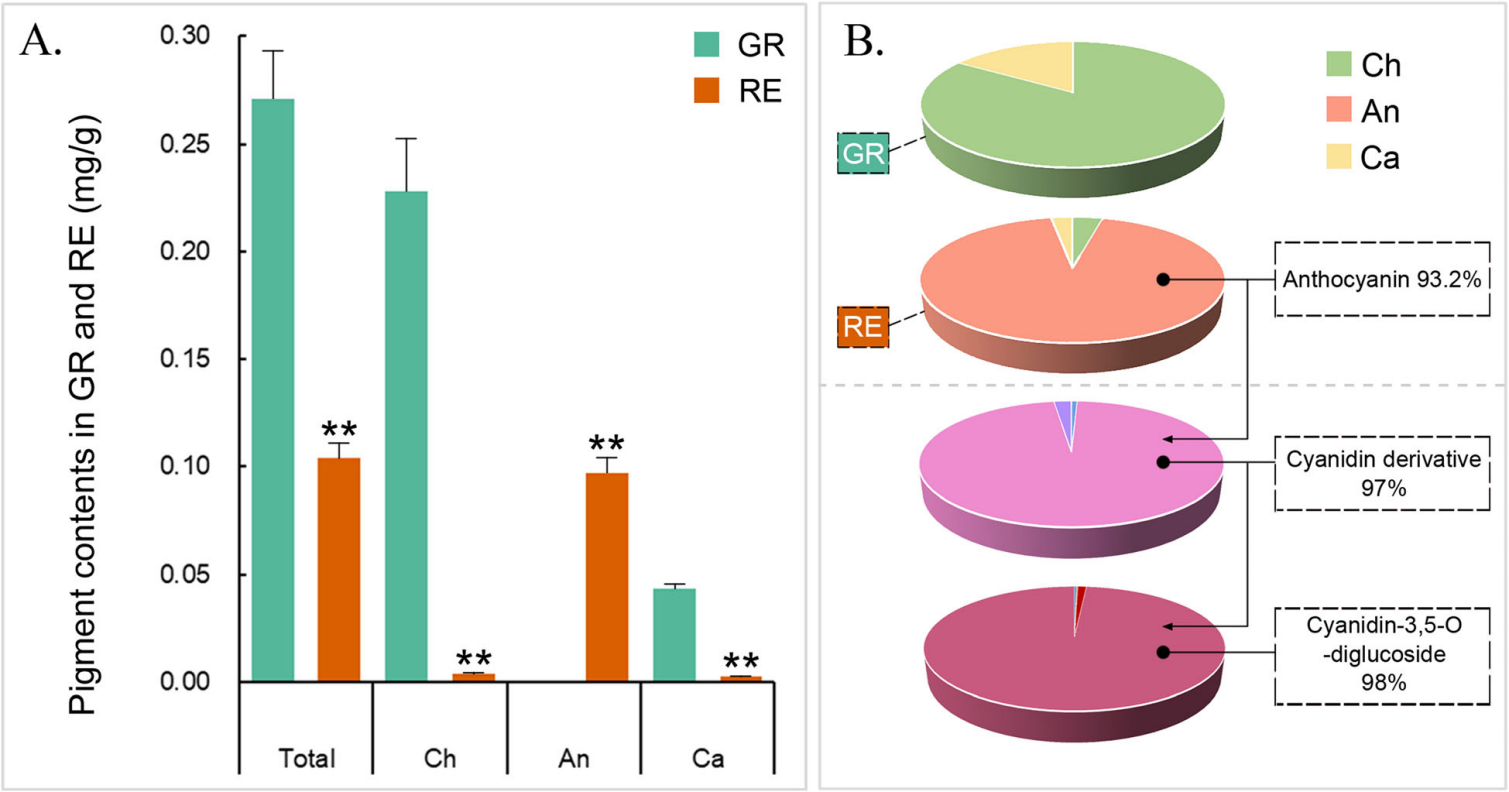
Pigment contents and relative proportions in GR and RE. **a** Content of pigments in GR and RE. **b** Relative proportion of pigments in GR and RE. Error bars represent ± SE of biological triplicates. Asterisks represent significant differences in gene expression between GR and RE parts (one asterisk for *P* < 0.05 and two asterisks for *P* < 0.01). Ch, chlorophyll. An, anthocyanin. Ca, carotenoid

**Fig. 4:**
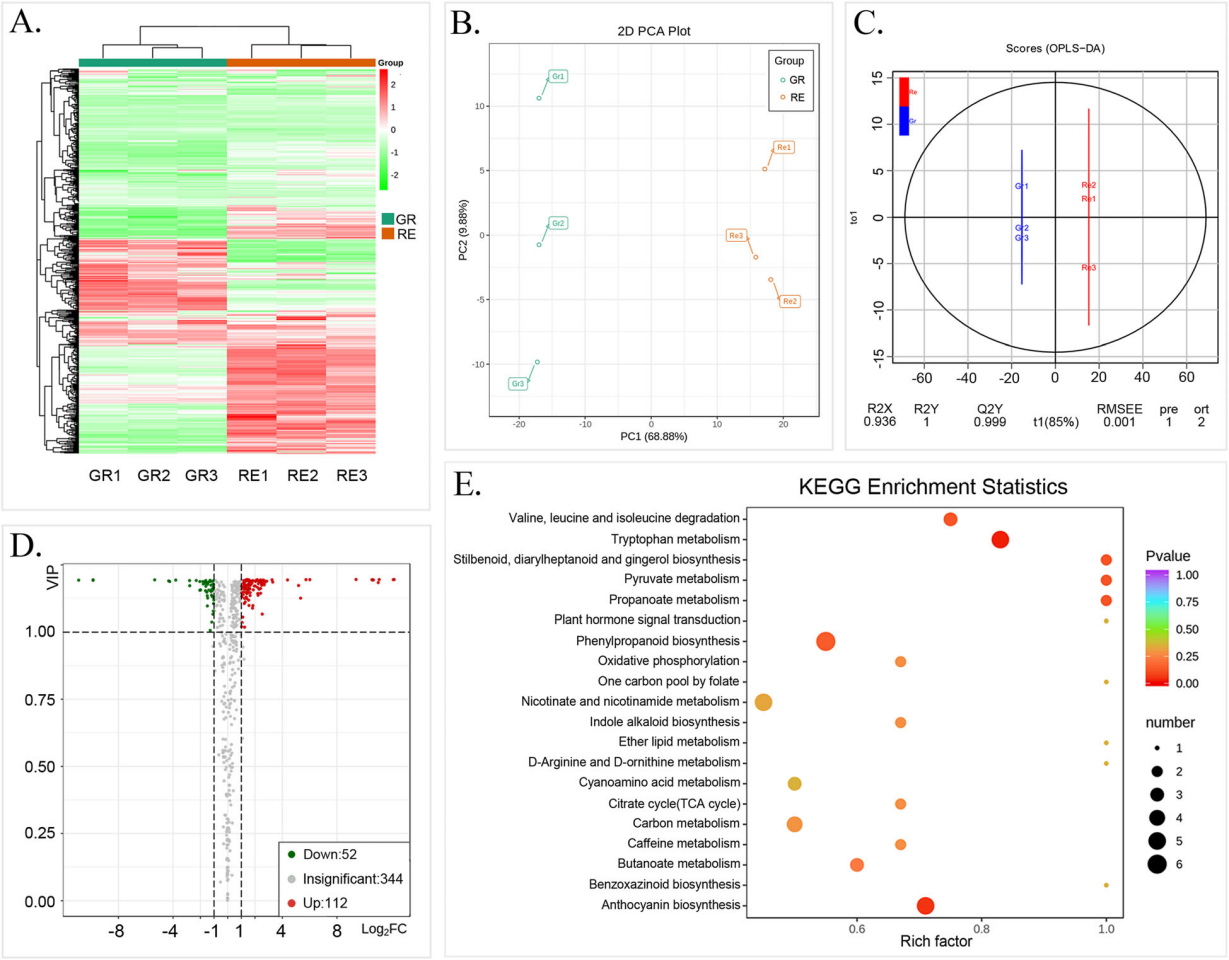
Metabolite profiles between GR and RE. **a** Heatmap and hierarchical clustering showing differentially produced metabolites (DPMs). **b** PCA plot. **c** OPLS-DA plot. **d** Volcano plots of upregulated and downregulated metabolites. **e** KEGG enrichment plot of DPMs

The pigment contents and relative proportions of GR and RE are shown in Fig. 3. The chlorophyll content in GR was approximately 57 times higher than that in RE, while anthocyanins were undetectable in GR (Fig. 3a). According to Fig. 3b, chlorophyll, accounting for 85.9% of all pigments, was the most prominent pigment in GR. The total pigment content of RE was less than half of the GR pigment content, with chlorophyll and carotenoid contents of no more than 0.05 mg/g. However, total anthocyanins accounted for 93.2% of the total pigments in RE, followed by a small proportion of chlorophyll and carotenoid. Meanwhile, the chlorophyll content in GR and anthocyanin content and RE samples both was correlated with their a* values respectively, but the other indexes were not. To determine the types of anthocyanins in RE, anthocyanin-related metabolites were analyzed by UPLC-ESI-MS/MS. First, a total of 508 metabolites were identified in GR and RE, and the most abundant metabolites were flavonoids, organic acids, alkaloids, lipids, amino acids and derivatives (Table S1). The cluster heatmap, PCA and OPLS-DA plots showed differences in metabolites between two phenotypes (Fig. 4a-c). After selecting differentially produced metabolites (DPMs) based on an absolute log_2_fold-change ≥1 among the total metabolites with a VI*P* value ≥1, 164 DPMs were filtered. The volcano plots indicated that there were 112 upregulated and 52 downregulated metabolites between the GR and RE samples (Fig. 4d). Additionally, 67 DPMs were annotated to the KEGG database, including “Metabolic pathways” (ko01100), “Biosynthesis of secondary metabolites” (ko01110), “Biosynthesis of animo acids” (ko01230), “ABC transporters” (ko02010), “Phenylpropanoid biosynthesis” (ko00940), “Flavonoid biosynthesis” (ko00941), “Anthocyanin biosynthesis” (ko00942), “Flavone and flavonol biosynthesis” (ko00944), “Aminoacyl-tRNA biosynthesis” (ko00970) and so on (Fig. 4e, Table S2, File S1). These DPMs respectively belonged to “Amino acids and derivatives”, “Phenolic acids”, “Nucleotides and derivatives”, “Flavonoids”, “Alkaloids”, “Organic acids”, “Lipids” and “Others”. Ten kinds of anthocyanins were detected, with 4 belonging to cyanidin type, 3 belonging to delphinidin type and others respectively belonging to malvidin type, pelargonidin type and peonidin type. In the anthocyanin-related pathways (ko00940, ko00941, ko00942), 15 metabolites including 6 kinds of anthocyanins showed significantly different production levels between GR and RE samples (Table 1). According to varieties of anthocyanin identified, we inferred that three typical biosynthetic branches of anthocyanins showed in ko00942 do exist in *A. comosus* var. *bracteatus*, and substrates were more catalyzed to form cyanidin-type derivatives. Among these anthocyanins, cyanidin derivatives, a category of pigments showing different degrees of red color, accounted for 97% of the total anthocyanin content (Fig. 3b), followed by extremely small rates of other types of anthocyanins. In particular, cyanidin-3,5-O-diglucoside (pme1777) accounted for 98% of cyanidin derivatives in RE. Although it was both detected in GR and RE, peak area value of this metabolite in RE reached up to 1.56E+ 08, which was 4.5 times higher than that of GR. These suggested that accumulated anthocyanins that peak area values exceeded a critical number between the range (3.47E+ 07 to 1.56E+ 08) probably can reach a visible level. Meanwhile, other 9 types of anthocyanins were all no more than minimum of the range, which indicated that cyanidin-3,5-O-diglucoside was the most prominent anthocyanin responsible for the RE phenotype.

**Table 1:** DPMs in anthocyanin-related pathways between GR and RE

Name	GR	RE	VIP	Log_2_FC	Type	Cpd_ID	Related Map
3-O-p-Coumaroyl quinic acid	3.69E+ 05	1.09E+ 06	1.19E+ 00	1.56E+ 00	up	C12208	ko00940, ko00941
Caffeic acid	1.29E+ 05	5.25E+ 06	1.13E+ 00	5.34E+ 00	up	C01197	ko00940
Ferulic acid	1.47E+ 05	4.23E+ 06	1.18E+ 00	1.52E+ 00	up	C01494	ko00940
Caffeic aldehyde	1.66E+ 05	5.33E+ 04	1.19E+ 00	–	down	C10945	ko00940
Chlorogenic acid	1.28E+ 06	4.04E+ 06	1.19E+ 00	1.65E+ 00	up	C00852	ko00940, ko00941
Coniferin	1.17E+ 06	2.60E+ 06	1.18E+ 00	1.15E+ 00	up	C00761	ko00940
Hesperetin	8.50E+ 03	4.14E+ 03	1.11E+ 00	–	down	C01709	ko00941
Apigenin-8-C-Glucoside	9.70E+ 06	4.63E+ 06	1.13E+ 00	–	down	C01460	ko00941
Delphinidin-3,5-O-diglucoside	1.74E+ 05	8.57E+ 05	1.19E+ 00	2.30E+ 00	up	C16312	ko00942
Delphinidin-3-O-glucoside	4.98E+ 05	2.71E+ 06	1.18E+ 00	2.45E+ 00	up	C12138	ko00942
Cyanidin-3-O-rutinoside	1.82E+ 05	1.73E+ 06	1.19E+ 00	3.25E+ 00	up	C08620	ko00942
Pelargonidin-3,5-diglucoside	1.93E+ 05	1.16E+ 06	1.19E+ 00	2.59E+ 00	up	C08725	ko00942
Malvidin-3,5-O-diglucoside	1.26E+ 04	2.57E+ 04	1.17E+ 00	1.03E+ 00	up	–	–
Cyanidin-3,5-O-diglucoside	3.47E+ 07	1.56E+ 08	1.19E+ 00	2.17E+ 00	up	C08639	ko00942

Columns 2 and 3 represent metabolite peak area values of different samples

### Differentially expressed genes (DEGs) in the GR and RE samples of *A. comosus* var. *bracteatus*

**Fig. 5:**
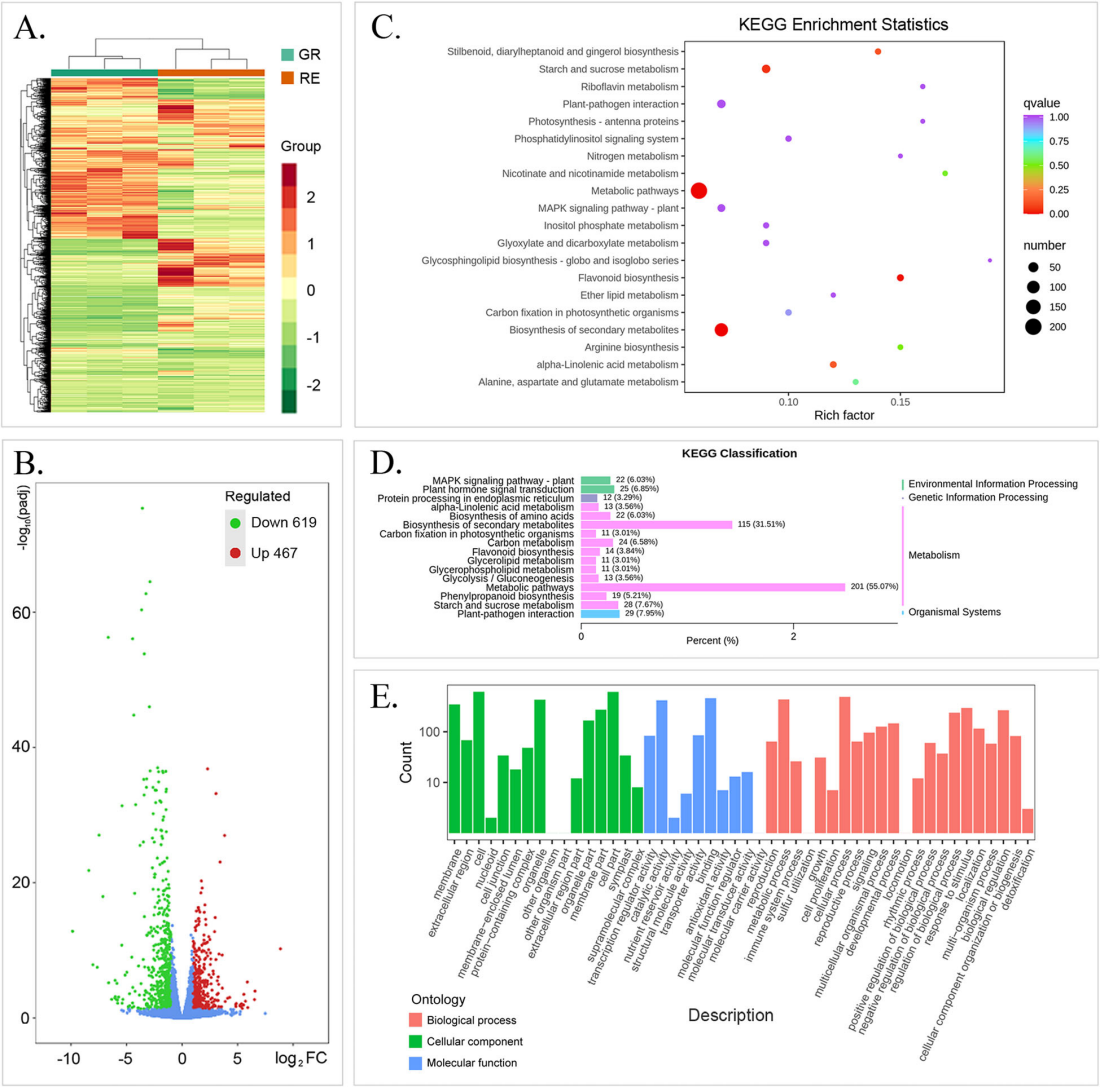
Gene expression and enrichment profiles of GR and RE samples of *A. comosus* var. *bracteatus*. **a** Heatmap and hierarchical clustering showing DEGs. **b** Volcano plots showing the number of DEGs in different regulation models. **c** KEGG pathway enrichment plots. **d** KEGG classification showing enriched pathways. **e** Number of DEGs in the GO classification

According to the reference transcriptome, 20,905 genes were obtained by using RNA-seq technology (Table S3). The clustering heatmap indicated that GR and RE had different gene expression patterns (Fig. 5a). Differentially expressed genes (DEGs) were selected by an absolute log_2_fold-change ≥1 with a false discovery rate (FDR) ≤ 0.05 and compared to six public databases (KEGG, NR, SwissProt, Tremble, GO and KOG) to determine their potential functions. In total, 1086 DEGs were selected, and volcano figures revealed that there were 619 downregulated and 467 upregulated DEGs between the GR and RE samples (Fig. 5b). A total of 365 DEGs in 121 KEGG pathways were enriched, which including “Metabolic pathways” (ko01100), “Biosynthesis of secondary metabolites” (ko01110), “Plant-pathogen interaction” (ko04626), “Starch and sucrose metabolism” (ko00500), “Plant hormone signal transduction”(ko04075), “Carbon metabolism”(ko01200), “Biosynthesis of amino acids”(ko01230), “MAPK signaling pathway-plant”(ko04016), “Phenylpropanoid biosynthesis”(ko00940), “Flavonoid biosynthesis”(ko00941), “Anthocyanin biosynthesis” (ko00942), “Glycolysis/Gluconeogenesis”(ko00010) and so on (Fig. 5c, d, Table S4, File S2). The GO bar plot indicated that the number of DEGs involved in metabolic process, cellular processes, cells, cell parts, catalytic activity, binding, etc. was significantly greater than 100 (Fig. 5e). In addition, 110 transcription factors (TFs) that were differently expressed in GR and RE were identified, belonging to WRKY, NAC, C2H2, bZIP, bHLH, MYB and other families (Table S5). Among these TFs, *MYB5* (Aco023263.1.path1) and *MYB82* (Aco023266.1.path1) were annotated as “transcription factor TT2” and “transcription factor TT2-like”. In order to compare different transcript levels of anthocyanin-related genes, 40 genes that had potential relationship with anthocyanin biosynthesis were selected for comparison between GR and RE and were shown in Table 2. Meanwhile, 11 of them were DEGs between GR and RE, and 9 of these DEGs were up-regulated, including 2 *CHI* (Aco012547.1.path1, Aco014232.1.path1), 1 *F3H* (Aco003857.1.path1), 1 *F3′H* (Aco003857.1.path1), 1 *DFR* (Aco006769.1.path1), 1 *3GT* (Aco012126.1.path1), 1 *ABCA3* (Aco006845.1.path1), 1 *MYB82* (Aco023266.1.path1) and 1 *MYB5* (Aco023263.1.path1). Among them, absolute values of log2FC of *F3H*, *DFR*, *MYB82* and *MYB5* all exceeded 2, which indicated that theses structure genes and TFs probably play important roles in differences of anthocyanin accumulation between GR and RE and are worthy of investigation on their relationship.

**Table 2:** Genes related to anthocyanin biosynthesis

Name	ID	GR	RE	Log_2_FC	*P* value	Type	KEGG_ID
*PAL*	Aco020618.1.path1	12.09	5.42	−1.283	4E-05	down	K10775
	Aco007727.1.path1	0.46	0.85	0.755	0.02258	–	
	Aco010091.1.path1	0.25	0.25	−0.159	0.82465	–	
	Aco013943.1.path1	45.43	87.97	0.845	0.00044	–	
*4CL*	Aco006521.1.path1	91.38	95.52	−0.058	0.74187	–	K01904
	Aco011626.1.path1	1.43	1.68	0.137	0.77702	–	
	Aco017002.1.path1	1.21	1.51	0.213	0.5899	–	
	Aco017294.1.path1	0.06	0	−1.935	0.6324	–	
	Aco019974.1.path1	1.09	1.79	0.618	0.41004	–	
	Aco019975.1.path1	45.62	51.09	0.043	0.78884	–	
	Aco025129.1.path1	30.5	53.74	0.698	0.28868	–	
*CHI*	Aco012547.1.path1	26.16	58.11	1.051	0.00038	up	K01859
	Aco014232.1.path1	45.47	113.57	1.217	2.9E-06	up	
	Aco022584.1.path1	452.78	448.54	−0.13	0.59715	–	
	Aco025098.1.path1	65.10	62.28	−0.176	0.28868	–	
*CHS*	Aco016200.1.path1	149.59	293.62	0.865	4.5E-06	–	K00660
*F3H*	Aco018609.1.path1	4.15	18.20	2.032	3.5E-11	up	K00475
	Aco027900.1.path1	0.11	0.12	−0.036	0.9743	–	
*F3′H*	Aco003857.1.path1	27.99	61.63	1.027	3.7E-13	up	K05280
	Aco003885.1.path1	11.03	23.60	0.982	4.2E-10	–	
	Aco003886.1.path1	0.05	0.28	2.272	0.35361	–	
*F3′5′H*	Aco017169.1.path1	5.70	7.98	0.371	0.12805	–	K13083
	Aco013254.1.path1	0.23	0.72	1.483	0.19733	–	
*DFR*	Aco006769.1.path1	3.51	16.73	2.151	1.8E-07	up	K13082
	Aco014886.1.path1	0.19	0.47	1.113	0.41798	–	
	Aco014887.1.path1	7.43	10.95	0.439	0.07125	–	
*FLS*	Aco000551.1.path1	0	0.04	2.554	0.25109	–	K05278
	Aco011051.1.path1	3.34	1.30	−1.475	0.00043	down	
	Aco001560.1.path1	20.24	23.25	0.078	0.73827	–	
	Aco002683.1.path1	15.29	25.77	0.642	0.00261	–	
	Aco007045.1.path1	0.03	0	−2.591	0.51669	–	
	Aco012471.1.path1	0.11	0.08	−0.565	0.73375	–	
*ANS*	Aco007046.1.path1	0.89	0.20	−2.26	0.00911	–	K05277
*3GT*	Aco012126.1.path1	20.75	87.45	1.973	1.6E-12	up	K12930
*ABCA3*	Aco006845.1.path1	13.99	38.12	1.324	4.9E-09	up	K05643
	Aco007263.1.path1	42.66	32.95	−0.506	0.22723	–	
	Aco008459.1.path1	23.40	25.61	0.014	0.92605	–	
	Aco014363.1.path1	9.14	11.88	0.259	0.22712	–	
*MYB82*	Aco023266.1.path1	0.25	1.69	2.666	0.00044	up	K09422
*MYB5*	Aco023263.1.path1	5.02	40.80	2.924	1.5E-15	up	

Columns 3 and 4 represent FPKM values of transcripts in GR and RE. Underline represents DEGs

### qPCR validation

**Fig. 6:**
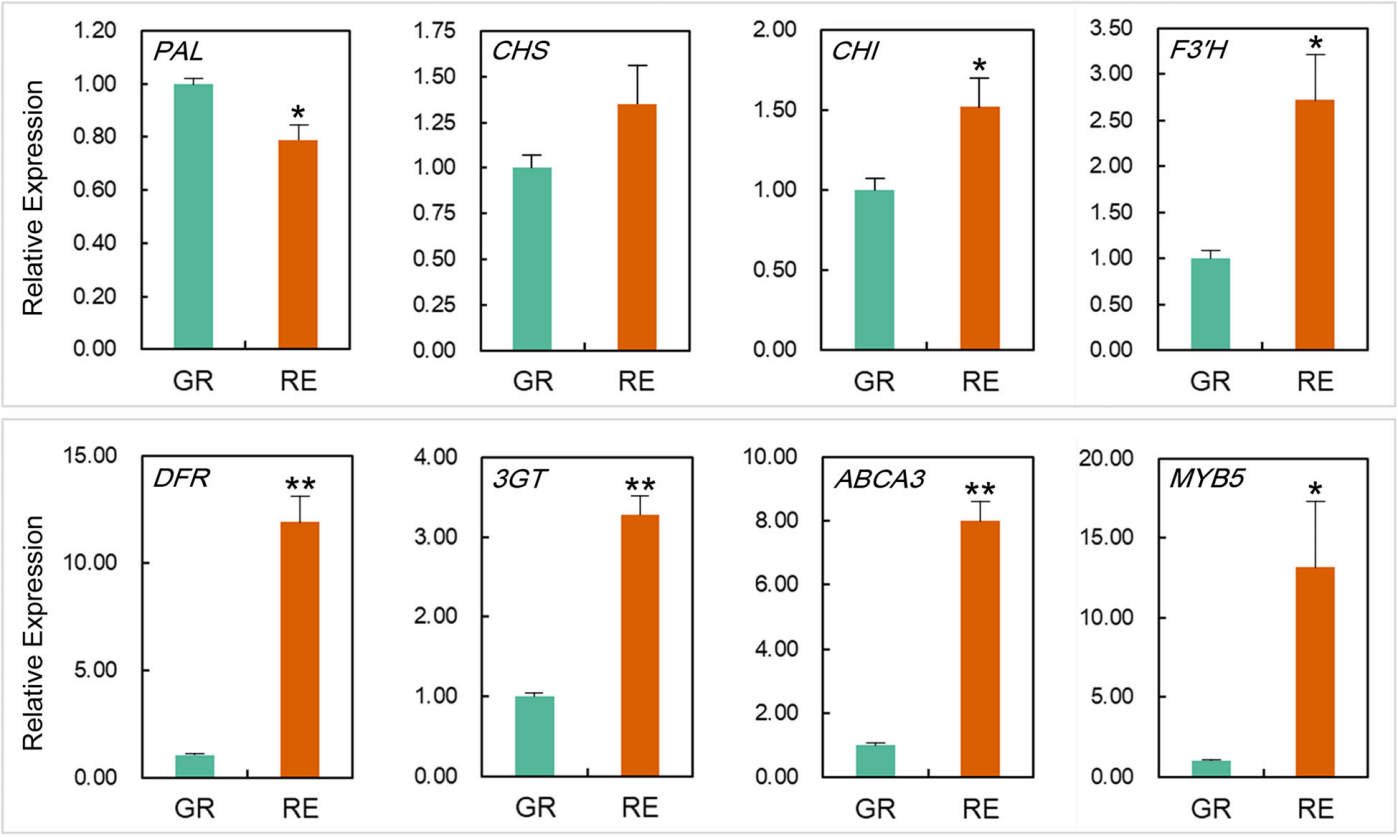
Transcriptional levels of eight selected genes related to anthocyanin biosynthesis between GR and RE samples. Error bars represent ± SE of biological triplicates. Asterisks represent significant differences in gene expression between GR and RE parts (one asterisk for *P* < 0.05 and two asterisks for *P* < 0.01)

To validate the RNA-Seq results, eight anthocyanin-related genes including *PAL* (Aco020618.1.path1), *CHS* (Aco016200.1.path1), *CHI* (Aco012547.1.path1), *F3′H* (Aco003857.1.path1), *DFR* (Aco006769.1.path1), *3GT* (Aco012126.1.path1), *ABCA3* (Aco006845.1.path1) and *MYB5* (Aco023263.1.path1) were selected, and their relative expression levels in the GR and RE phenotypes were analyzed by using qPCR. The expression patterns of these genes are generally consistent with the RNA-Seq results (Fig. 6).

### Correlation analysis of the relationship between genes and metabolites involved in anthocyanin biosynthesis of *A. comosus* var. *bracteatus*

**Fig. 7:**
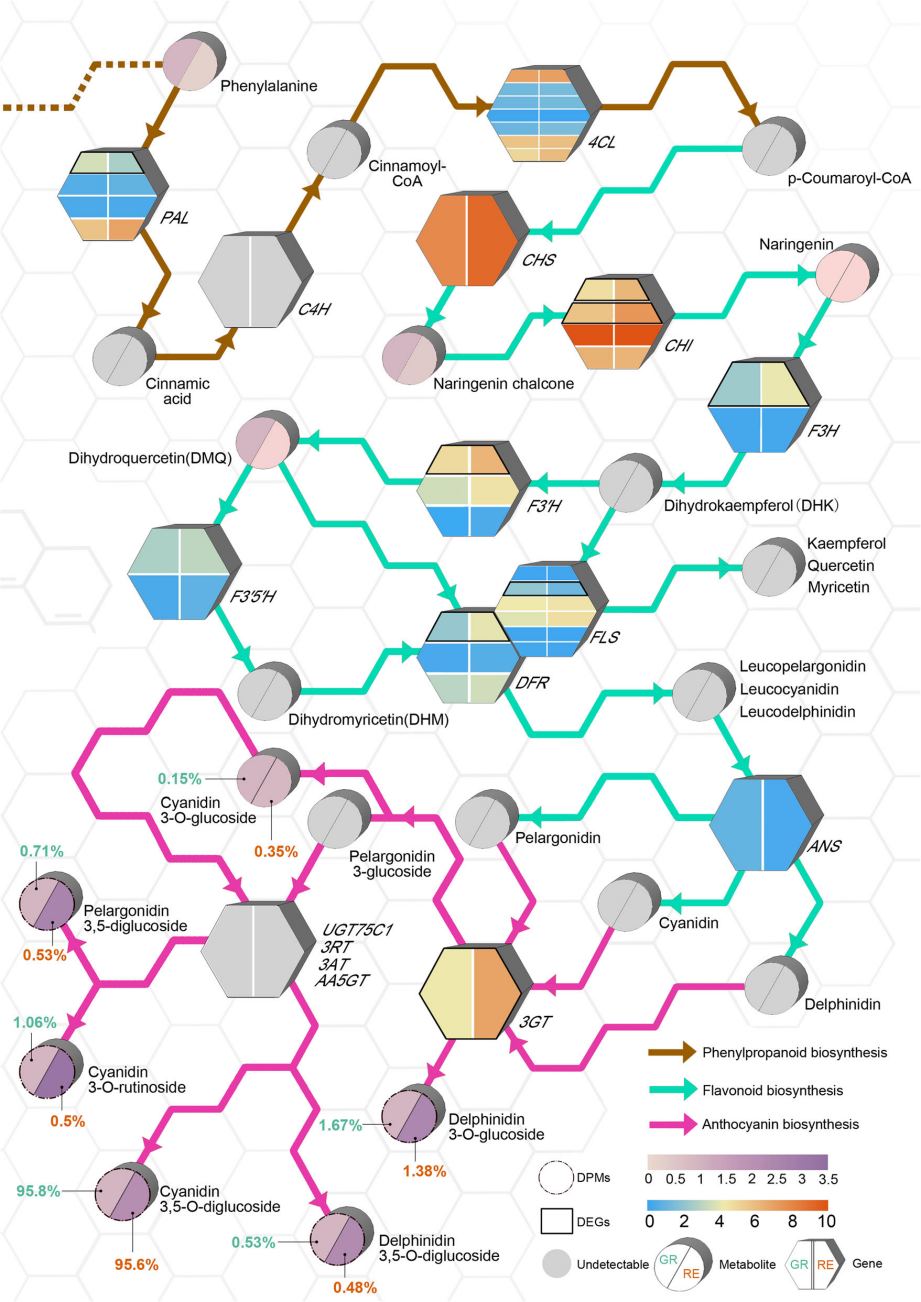
Metabolites and genes involved in the biosynthesis of anthocyanin in *A. comosus* var. *bracteatus*. Anthocyanins were synthesized through three pathways: phenylpropanoid biosynthesis, flavonoid biosynthesis and anthocyanin biosynthesis. The color scale of metabolites indicates the log_2_FC between the two types of samples. The color scale of genes indicates the log_2_ transformation value of FPKM. The percentage represents the ratio of anthocyanins among all detected anthocyanins in GR and RE. The gray circles and hexagons indicate undetectable metabolites and genes, respectively

In this study, a pathway map containing metabolites and structural genes related to anthocyanin biosynthesis was constructed based on the metabolome and transcriptome analyses with the KEGG database (Fig. 7). Metabolites and genes were labeled in different colors, indicating production and expression levels between GR and RE. The expression of 1 *PAL* (Aco020618.1.path1) and 1 *FLS* (Aco011051.1.path1) in GR was significantly higher than that in RE. In contrast, expression of 2 *CHIs* (Aco012547.1.path1 and Aco014232.1.path1), 1 *F3H* (Aco018609.1.path1), 1 *F3′H* (Aco003857.1.path1), 1 *DFR* (Aco006769.1.path1) and 1 *3GT* (Aco012126.1.path1) in RE was significantly higher than that in GR. However, early metabolites such as naringenin chalcone (pme2960), naringenin (pme0376) and dihydroquercetin (DHQ, mws0044) did not show significant differences between GR and RE. In the late stage of anthocyanin biosynthesis, the production level of pelargonidin-3,5-diglucoside (pme1793), cyanidin-3-O-rutinoside (pme1773), cyanidin-3,5-O-diglucoside (pme1777), delphinidin-3,5-O-diglucoside (pmf0116) and delphinidin-3-O-glucoside (pme1398) in RE was significantly higher than that in GR. In particular, cyanidin-3,5-O-diglucoside accounted for 95.6% of all detected anthocyanins in RE, followed by other types of anthocyanins, which were present only at extremely low levels. Additionally, the existence of delphinidin derivatives suggested that purple or blue colors did exist in RE but were probably masked by the high proportion of cyanidin derivatives.

**Fig. 8:**
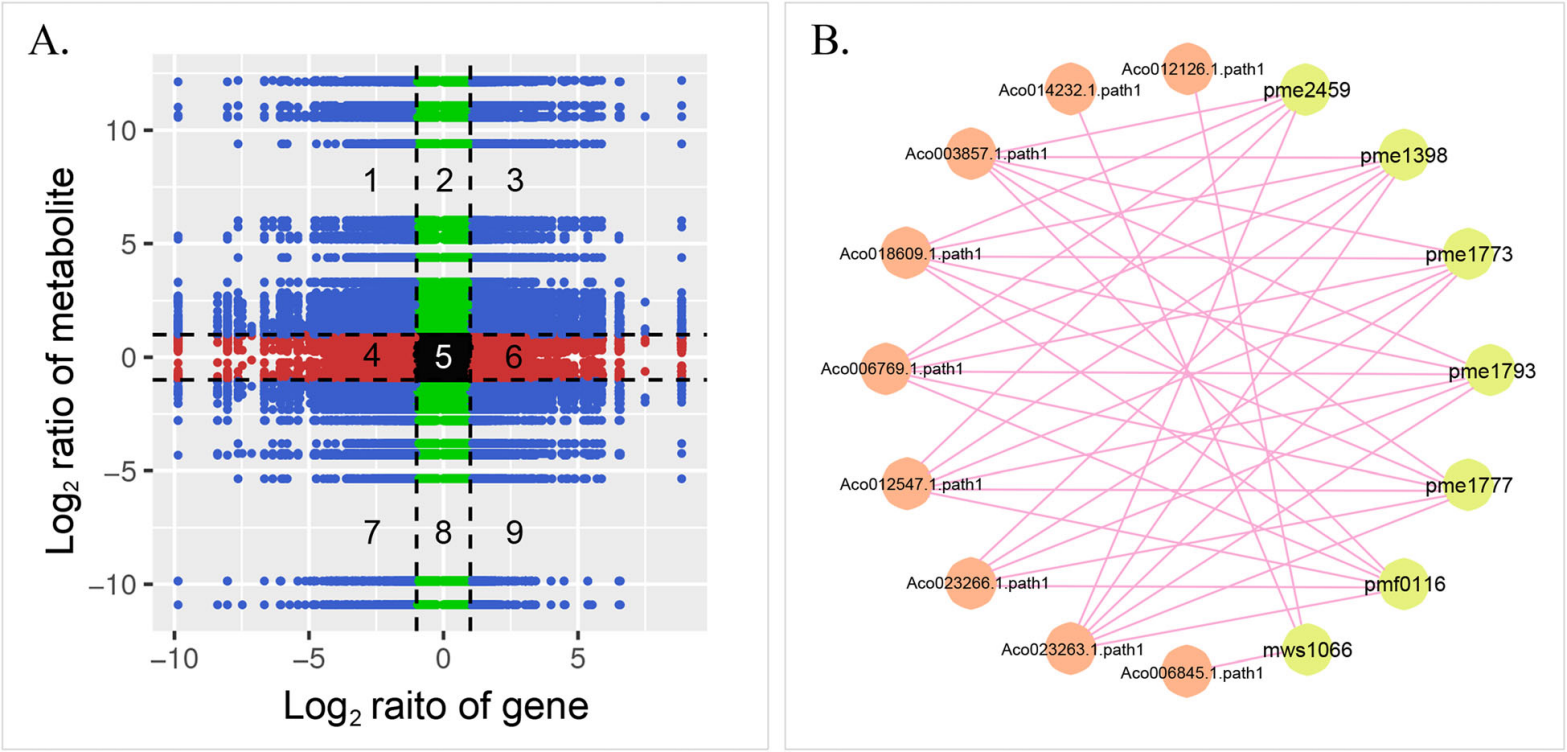
Profiles of the correlation analysis of anthocyanin-related genes and metabolites. **a** Nine-quadrant flat. **b** Correlations between anthocyanin-related genes and metabolites

To understand the relationship between anthocyanin-related genes and metabolites, correlation tests were carried out. In the nine-quadrant flat, metabolites and genes in the third and seventh quadrants showed consistent changes (Fig. 8a, Table S6). The nine-quadrant results showed that 1734 genes that had Pearson correlation coefficient (PCC) values ≥0.8, and 168 metabolites were identified in the third and seventh quadrants. Among these genes, 9 anthocyanin-related genes were identified and showed correlations with 7 anthocyanin-related metabolites in the third quadrant (Fig. 8b). *CHI* (Aco012547.1.path1), *F3′H* (Aco003857.1.path1), *F3H* (Aco018609.1.path1), *DFR* (Aco006769.1.path1) and *MYB5* (Aco023263.1.path1) were positively correlated with the content of luteolin-7-O-glucoside (pme2459), delphinidin-3-O-glucoside (pme1398), cyanidin-3-O-rutinoside (pme1773), pelargonidin-3,5-diglucoside (pme1793), cyanidin-3,5-O-diglucoside (pme1777) and delphinidin-3,5-O-diglucoside (pmf0116). *MYB82* (Aco023266.1.path1) were positively correlated with the content of delphinidin-3-O-glucoside, cyanidin-3-O-rutinoside, cyanidin-3-O-rutinoside, cyanidin-3,5-O-diglucoside and delphinidin-3,5-O-diglucoside. Naringenin-7-O-rutinoside (mws1066) was positively correlated with *CHI* (Aco014232.1.path1), *3GT* (Aco012126.1.path1) and *ABCA3* (Aco006845.1.path1).

## Discussion

### CIELAB values are closely associated with the Leaf Organization structures and pigment distribution of *A. comosus* var. *bracteatus*

The plant coloration shown in nature is not only attributed to different kinds and proportions of pigments distributed in plant organs but is also attributed to the internal structures of plant organs. As shown in Fig. 1, GR had a lower L* value than RE, which seemed to be related to a dark phenotype even when the chlorophyll content of GR far exceeded that of RE. Connected with leaf organization structures, this result might be related to the thickness of the GR leaf areas, which are much thicker than the RE areas. Therefore, it was not difficult to infer that the thicker the leaf tissue, the more mesophyll cells exist. Moreover, the cell shape and cell permeability in leaf tissues can be quite different, which easily leads to refraction and reflection of light [37–39]. Additionally, the physical green color itself is a mix of cyan and yellow colors, which are among the three primary colors in the CMYK color model. With the increase in tissue thickness, layers of green color tended to become dark, which also made the L* value of GR low. In contrast, the RE leaf areas were much thinner than the GR areas, and the mesophyll cells were colorless, with a few cells having anthocyanin in the epidermis. Thus, there was a relatively lower chance of light refraction and reflection in RE. In addition, different from the green color, the red color itself is one of three primary colors. The L* value of RE was much higher than that of GR even when the thickness of the colorless mesophyll tissue reached a certain level. This result suggested that the RE phenotype was not mainly influenced by the middle transparent cells but was mainly related to the anthocyanin content in the epidermis.

The b* values of GR and RE were not significantly different, and both were light yellow. The results suggested that these tissues were similar in some respects. According to Fig. 2, we inferred that the yellow color of both GR and RE was probably derived from the keratinized cell walls of epidermal cells, and the specialized cell wall structure is typical for plants under long-term environmental stress. To reduce the evaporation of water and adapt to the high-temperature environment, tropical plants such as *A. comosus* var. *bracteatus* have to develop a series of features to overcome these challenges. Thick wall tissue, such as keratinized cell walls, is a tropical plant feature that protects the water in plants from excessive evaporation [40]. From the chemical composition perspective, the thick-walled tissue mainly contains fatty acids that have hydrophobic functions. Therefore, we infer that the epidermis of both GR and RE showed slight coloration when certain thresholds were reached for fatty acid levels and tissue thickness.

### Flavonoids and phenolic acids are differentially produced in chimeric leaves of *A. comosus* var. *bracteatus*

Among 508 DPMs, the most abundant metabolites were flavonoids, according to the Table S1, and most of them were modified as varieties of glycosides, especially glucoside-type and diglucoside-type. We inferred that this result probably had relationship with storage and utilization of starch and sucrose because of different production of starch in GR and RE [41], which indicated that RE has function of storage and hydrolysis of starch necessary for photosynthesis in GR. Actually, some flavonoids also possess colors and show similar features with common pigments. For example, flavonoids were detected in the black and red rice, but the prominent pigment in red variety was not anthocyanin but flavan-3-ols oligomers, which is a kind of flavonoid [42]. This result indicated that variable glycogen-type flavonoids in *A. comosus* var. *bracteatus* may also influence the phenotype of red samples.

According to the metabolome analysis of *A. comosus* var. *bracteatus*, the peak areas of pelargonidin derivatives, cyanidin derivatives and delphinidin derivatives were detected in both GR and RE. Moreover, *F3H*, *F3′H* and *F3′5′H* had certain FPKM values in samples of the two phenotypes, which suggested that the three branches of different anthocyanidin biosynthesis exist in *A. comosus* var. *bracteatus*. According to previous reports, substrate specificity of DFR was confirmed as a key step for regulating anthocyanin types [43–45], and this provides a possible approach for regulating the leaf colors of *A. comosus* var. *bracteatus* by biotechnology.

Another interesting point is that 31 out of 36 metabolites belonging to phenolic acids were significantly up-produced in RE samples. Previous studies showed that phenolic acid, as a signal molecule in plants, play an important role in plant defense [46]. Meanwhile, phenolic acids, deriving from glycolysis and pentose phosphate pathway, are finally formed through a series of pathways including phenylpropanoid biosynthesis, which is also the anthocyanin-related pathway [47, 48]. Although there is no obvious evidence showing competence of substrates between biosynthesis of flavonoids and phenolic acids, functions of metabolites in these two categories are important for subsequent studies.

### Anthocyanin accumulation and distribution are probably influenced by a series of complicated processes in chimeric leaves of *A. comosus* var. *bracteatus*

In the annotated KEGG maps, not only structure genes in anthocyanin-related pathways but also in other pathways showed different expression between GR and RE samples, which indicated that differences of anthocyanin accumulation probably also have relationships with other metabolic pathways in chimeric leaves of *A. comosus* var. *bracteatus*. First, DEGs annotated in “Starch and sucrose metabolism”, “Glycolysis/Gluconeogenesis” and “Carbon metabolism” indicated that utilization of carbohydrates was different in GR and RE, and previous reports have showed the importance of sugars in the anthocyanin biosynthesis [49, 50]. DEGs in “Plant hormone signal transduction”, “MAPK signaling pathway-plant” and “Plant-pathogen interaction” indicated that a series of genes, related to hormone responsiveness and adversity-stress responsiveness, were up-regulated in RE. Based on these results, we inferred that RE samples probably were more sensitive and vulnerable to environmental stimulations and changes.

From the aspect of differentially expressed TFs, MYB family was the most abundant family among them. MYB family has been reported to be involved in regulating anthocyanin biosynthesis [4, 51, 52], which controls anthocyanin biosynthesis genes mainly by two ways: MYB proteins interact with bHLH and WD40 proteins to form a MBW protein complex and then activate structural genes; and early biosynthetic genes (*CHS*, *CHI*, *F3H*, *F3′H*, *FLS*) that produce flavonols are regulated by independent MYB regulators (MYB11, MYB12) [53]. In the transcriptome files of *A. comosus* var. *bracteatus* (Table S5), two MYB genes, *MYB5* (Aco023263.1.path1) and *MYB82*(Aco023266.1.path1), were annotated as “transcription factor TT2 (A)” and “transcription factor TT2-like (A)”, respectively, which suggested that they probably have similar functions as *TT2* [54, 55]. Besides, there are some factors induced by hormones and stress, such as WRKYs, MADSs, NACs, AP2/ERF and so on. These factors can be stimulated by environmental change to target *cis*-elements in promoters of structural genes to regulate biological process including anthocyanin-related pathways.

Generally, these results suggested that specific anthocyanin accumulation and distribution in *A. comosus* var. *bracteatus* are more complicated than previous reports. Because of the chimeric trait, differences of anthocyanin production in two parts cannot be mechanically owing to anthocyanin-related genes differentially expression. Actually, the most enriched DPMs and DEGs distributed in many other pathways, such as biosynthesis of amino acids and aminoacyl-tRNA, signal transduction, metabolism of glucose and so on, which have correlation with physiological processes. The relationship between these physiological processes and anthocyanin biosynthesis in *A. comosus* var. *bracteatus* is unknown, and mechanisms behind these genes are worthy of further study.

## Conclusions

Analyses at the phenotypic, cellular and molecular levels showed that (1) CIELAB color values are closely associated with leaf tissue structures and anthocyanins hardly accumulate in green tissues but clearly accumulate in epidermal cells in the RE areas of *A. comosus* var. *bracteatus* leaves. (2) Ten different types of anthocyanins belonging to the three branches of the anthocyanin biosynthesis pathway were detected in both GR and RE samples, and cyanidin-3,5-O-diglucoside accounted for the highest proportion in RE and was present in significantly higher levels than those in GR. (3) *MYB5* and *MYB82* are two probable transcription factors that result in the specific accumulation and distribution of anthocyanin in different parts of *A. comosus* var. *bracteatus* leaves.

## Materials and methods

### Plant materials and sample preparation

**Fig. 9:**
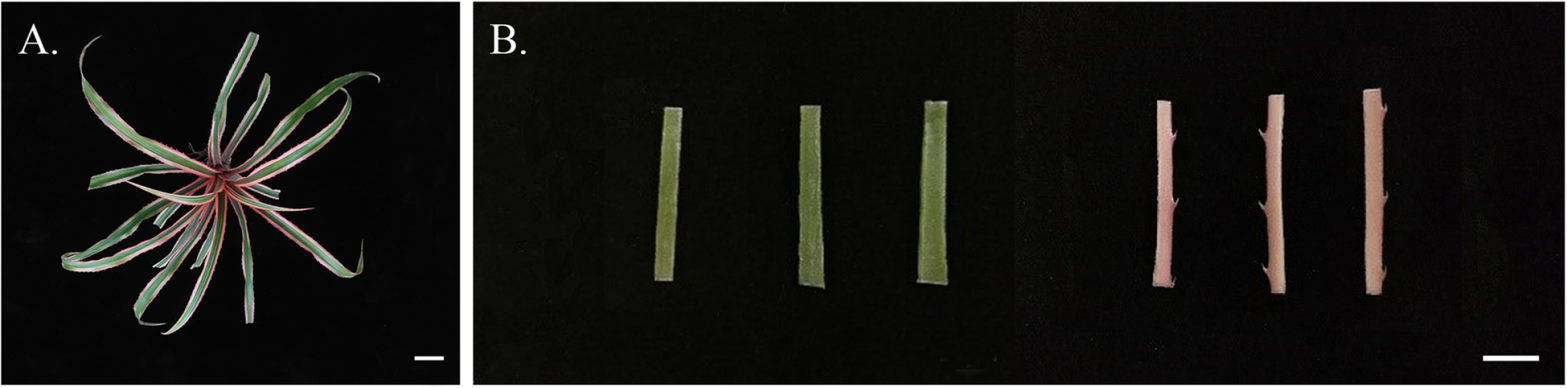
Chimeric leaves of *Ananas comosus* var. *bracteatus* used in this study. **a** *A. comosus* var. *bracteatus* chimeric plant. Bar = 5 cm. **b** Green central parts (GR) and red edges (RE) from chimeric leaves. Bar = 1 cm

The chimeric leaves of two-year-old *A. comosus* var. *bracteatus* from an experimental field at Sichuan Agricultural University were used in this study. Middle parts of chimeric leaves were collected from five plants and divided into two types of samples based on their colors: the green central parts (GR) and the red edges (RE) (Fig. 9), which facilitated following assays required for three biological replicates. After being washed completely, the two types of samples were collected for color estimation, pigment content determination and anatomical cross sectioning. Additionally, samples were cut into small pieces and immediately frozen with liquid nitrogen. The frozen samples were stored at − 80 °C for further transcriptome and metabolome analyses.

### Leaf color estimation

In this assay, a CM-2600d color analyzer (Konica Minolta CM-2600d, Japan) was used for color digitization. The measurement was taken on the surfaces of middle typical parts of chimeric leaves from plants, and both surfaces of each sample were measured for at least three times. The measurement of leaf color was based on the Commission Internationale de l’Éclairage (CIE D65/10°) scale [56, 57].CIE color indexes include the lightness component L* and two chromatic components a* and b*. L* values from 0 to 100 indicate the range from black to white; values from -a* to +a* represent a decrease in green and an increase in red; values from -b* to +b* represent a decrease in blue and an increase in yellow. In addition, C* represents chroma, which is calculated by the following equation: C* = (a*^2^ + b*^2^)^1/2^ [58]. The values of color were visualized by Photoshop 5.0.

### Anatomical cross sectioning

To obtain a better understanding of the anthocyanin distribution in the tissues of GR and RE samples, the samples were cut into blocks of an appropriate size for freehand cross sectioning. Blocks were further cut by a pair of overlapping blades, and a section from the gap between the blades was immediately transferred onto a glass slide sealed with a cover glass. Sections were observed by a microscope (XSP-15CA, Shanghai CSOIF Co., Ltd) and photographed by Cellsens Standard software.

### Chlorophyll and carotenoid content determination

The chlorophyll and carotenoid contents of GR and RE samples were determined. A sample of 0.1 g was cut into slices and transferred into a 15 mL centrifuge tube with 5 mL 95% ethanol for extraction. The tube was kept in the dark until the samples became colorless. Chlorophyll and carotenoid absorption was measured at 665, 649 and 470 nm with a UV-Vis spectrophotometer (UV1901 s/UV1901PCS, Shanghai Youke Instrument Co., Ltd.) equipped with 1.0 cm quartz cells, and the concentration was calculated following the method of Arnon [59].

### Anthocyanin content determination

The total anthocyanin contents of GR and RE samples were measured and calculated by using a pH differential protocol [60, 61].

### Sample extraction

Assays and analysis of metabolome followed the methods of Chen et al. [62], with some minor modifications. Freeze-dried leaves stored at − 80 °C were crushed using a mixer mill (MM 400, Retsch) with a zirconia bead for 1.5 min at 30 Hz. Then, 100 mg of the powder was weighed and extracted overnight at 4 °C with 0.6 ml 70% aqueous methanol. Following centrifugation at 10,000 g for 10 min, the extracts were absorbed (CNWBOND Carbon-GCB SPE Cartridge, 250 mg, 3 ml; ANPEL, Shanghai, China, www.anpel.com.cn/cnw) and filtrated (SCAA-104, 0.22 μm pore size; ANPEL, Shanghai, China, http://www.anpel.com.cn/) before the UPLC-MS/MS analysis.

### UPLC conditions

The sample extracts were analyzed using a UPLC-ESI-MS/MS system (UPLC, Shim-pack UFLC SHIMADZU CBM30A system, www.shimadzu.com.cn/; MS, Applied Biosystems 4500 Q TRAP, www.appliedbiosystems.com.cn/).The analytical conditions were as follows: The analytical conditions for the UPLC analysis included an Agilent SB-C18 column (1.8 μm, 2.1 mm*100 mm), and the mobile phase for solvent A consisted of pure water with 0.1% formic acid and for solvent B consisted of acetonitrile. Solvent system, water (0.04% acetic acid): acetonitrile (0.04% acetic acid); gradient program, 100:0 V/V at 0 min, 5:95 V/V at 20.0 min, 5:95 V/V at 22.0 min, 95:5 V/V at 22.1 min, 95:5 V/V at 28.0 min; flow rate, 0.25 ml min^− 1^; temperature, 40 °C; injection volume: 4 μl. The effluent was alternatively connected to an ESI-triple quadrupole-linear ion trap (Q TRAP)-MS.

### ESI-QTRAP-MS/MS

Triple quadrupole (QQQ) scans were acquired on a triple quadrupole-linear ion trap mass spectrometer (Q TRAP) and API 4500 Q TRAP UPLC/MS/MS System equipped with an ESI Turbo Ion-Spray interface. The system was operated in positive and negative ion mode and controlled by Analyst 1.6.3 software (AB Sciex). The ESI source operation parameters were as follows: ion source, turbo spray; source temperature, 550 °C; ion spray voltage (IS), 5500 V (positive ion mode)/− 4500 V (negative ion mode); ion source gas I (GSI), gas II (GSII), curtain gas (CUR), 50, 60, and 30.0 psi, respectively; collision gas (CAD), high. Instrument tuning and mass calibration were performed with 10 and 100 μmol/L polypropylene glycol solutions in QQQ modes, respectively. QQQ scans were acquired via MRM experiments with collision gas (nitrogen) set to 5 psi. DP and CE for individual MRM transitions were performed with further DP and CE optimization. A specific set of MRM transitions were monitored for each period according to the metabolites eluted within this period [63].

### RNA library preparation for transcriptome sequencing

A total amount of 3 μg RNA per sample was used as input material for the RNA sample preparations. Sequencing libraries were generated using NEBNext® Ultra™ RNA Library Prep Kit for Illumina® (NEB, USA) following the manufacturer’s recommendations, and index codes were added to attribute sequences to each sample. Briefly, mRNA was purified from total RNA using poly-T oligo-attached magnetic beads. Fragmentation was carried out using divalent cations under elevated temperature in NEBNext First Strand Synthesis Reaction Buffer (5X). First-strand cDNA was synthesized using a random hexamer primer and M-MuLV Reverse Transcriptase (RNase H^−^). Second-strand cDNA synthesis was subsequently performed using DNA Polymerase I and RNase H. Remaining overhangs were converted into blunt ends via the exonuclease/polymerase activities. After adenylation of the 3′ ends of DNA fragments, NEBNext Adaptor with a hairpin loop structure was ligated to prepare for hybridization. To select cDNA fragments with lengths of 150 ~ 200 bp (preferentially), the library fragments were purified with AMPure XP system (Beckman Coulter, Beverly, USA). Then, 3 μl USER Enzyme (NEB, USA) was used with size-selected, adaptor-ligated cDNA at 37 °C for 15 min followed by 5 min at 95 °C before PCR. Then PCR was performed with Phusion High-Fidelity DNA polymerase, Universal PCR primers and Index (X) Primer. Finally, PCR products were purified (AMPure XP system) and library quality was assessed on the Agilent Bioanalyzer 2100 system. The clustering of the index-coded samples was performed on a cBot Cluster Generation System using TruSeq PE Cluster Kit v3-cBot-HS (Illumina) according to the manufacturer’s instructions. After cluster generation, the library preparations were sequenced on an Illumina HiSeq platform and 125 bp/150 bp paired-end reads were generated. Quality-filtered Illumina reads of the reference data are available in the NCBI BioProject database (https://www.ncbi.nlm.nih.gov/bioproject/?term=PRJEB33121) [64].

### Gene annotation and differential gene expression analysis

All expressed genes were functionally annotated using the following databases: KEGG, Nr, Swiss-Prot, Tremble, KOG, GO, and Pfam. Gene expression levels were measured using FPKM (fragments per kilobase of transcript per million mapped reads) values based on the number of uniquely mapped reads [65]. For genes with more than one alternative transcript, the longest transcript was selected to calculate the FPKM value. Differential expression analysis of the three groups was performed using the DESeq R package (1.22.2). DESeq provides statistical routines for determining differential expression in digital gene expression data using a model based on the negative binomial distribution. The resulting *P* values were adjusted using the Benjamini-Hochberg approach for controlling the false discovery rate (FDR). Genes with an adjusted *P* value < 0.05 found by DESeq were considered differentially expressed.

### Validation of RNA-seq data by qPCR

Total RNA was reverse transcribed to generate first strand cDNA using an Evo M-MLV RT for PCR kit (Accurate Biotechnology, Hunan, Co., Ltd.). The *α-tubulin* was used as an internal control for normalization [66]. qPCR was performed on a qPCR instrument (BIORAD CFX96 real-time PCR detection system, America), and real-time PCRs were performed using a SYBR® Green Premix Pro TaqHS qPCR kit (Accurate Biotechnology, Hunan, Co., Ltd.). The 2^−ΔΔCT^ method [67] was used for quantitative analysis of the relative expression levels. The primers used in the qPCR analysis are shown in Table 3.

**Table 3:** qPCR primer sequences of eight genes

Gene Name and ID	F Primer (5′-3′)	R Primer (5′-3′)
*PAL* (Aco020618.1.path1)	TGAAGTCCGCCGTCAAGA	GCTCCTCCCTAACGAACCTGTA
*CHS* (Aco016200.1.path1)	GTGCCGCTGCTGTTATCC	TGTTCCCGAACTCGCTCA
*CHI* (Aco012547.1.path1)	GATTCCTTCTTTGAGGCACT	GTTTGGAAAAACTCGGCAAT
*F3′H* (Aco003857.1.path1)	GGGGAGGATTACAGAAACG	CCCTTGAGGTCCACATTTT
*DFR* (Aco006769.1.path1)	AGCATCATCCCACCTCTTG	TCGTCGTCATCGGTATCAG
*3GT*(Aco012126.1.path1)	AGCACGTTCGGCTTAGGAC	AGAGGCAGTTGCTCGGGTC
*ABCA3*(Aco006845.1.path1)	AGGCGATGGGTTGGCTATGC	AGAAGAAAGTTGCGGCGAGA
*MYB5*(Aco023263.1.path1)	AGATGAGACCAAGAACCCAC	CCCGCTATCAAAGACCAC

### Correlation analysis between Metabolome and Transcriptome data

Pearson correlation coefficients were calculated for metabolome and transcriptome data integration. In this study, log conversion of data was performed uniformly before analysis. For the joint analysis between the metabolome and transcriptome, the cor program from R was used, and the screening criterion was a Pearson correlation coefficient (PCC) > 0.8.

### Statistical data analysis

Digital results of leaf color estimation, pigment determination and qPCR were statistically compared by *t*-test in the SPSS.22 software. Analyst 1.5 software were used in data acquired in the QQQ experiment and peak areas were integrated using the IntelliQuan algorithm. Results of PCA and PCC were statistically obtained using R (base package, version 3.5.0). Pheatmap was adopted for the hierarchical clusters.

## Supplementary Information

**Additional file 1: File S1**: Annotated KEGG maps of metabolites. Blue plots indicate no significant changes between GR and RE samples. Red/green plots indicate up/down production of metabolites in RE samples compared with GR samples. White plots indicated undetectable metabolites.

**Additional file 2: File S2**: Annotated KEGG maps of genes. Blue bars indicate no significant changes between GR and RE samples. Red/green bars indicate up/down regulation of genes in RE samples compared with GR samples. White bars indicated undetectable genes.

**Additional file 3: Table S1**: Characteristics of all detected metabolites in GR and RE samples of *Ananas comosus* var. *bracteatus* chimeric leaves.

**Additional file 4: Table S2**: Numbers of Annotated metabolites and DPMs distributing in KEGG classification.

**Additional file 5: Table S3**: Characteristics of all genes in GR and RE samples of *Ananas comosus* var. *bracteatus* chimeric leaves.

**Additional file 6: Table S4**: Numbers of Annotated genes and DEGs distributing in KEGG classification.

**Additional file 7: Table S5**: Differentially expressed transcription factors between GR and RE samples.

**Additional file 8: Table S6**: Correlation analysis results of Metabolites and genes in the third and seventh quadrants of nine-quandrant flat.

## Data Availability

The transcriptional datasets analyzed during the current study are available in the National Center for Biotechnology information (NCBI) repository, and the BioProject accession number was PRJNA720713 (https://www.ncbi.nlm.nih.gov/bioproject/?term=PRJNA720713). Other data supporting the results of this article was included within the article and supplementary information.

## Abbreviations

bHLH: Basic helix-loop-helix
CHS: Chalcone synthesis
F3H: Flavanone 3-hydroxylase
F3′H: Flavonoid 3′-hydroxylase
F3′5′H: Flavonoid 3′5′-hydroxylase
DFR: Dihydroflavonol 4-reductase
ANS: Anthocyanidin reductase
3GT: UDP-Glucose: Flavonoid 3-O-glucosyltransferase
GST: Glutathione S-transferase
MRP: Multidrug resistance-associated protein
MATE: Multidrug and toxic compound extrusion
TT2: TRANSPARENT TESTA 2
CAM: Crassulacean acid metabolism
RHSCC: Royal horticulture society color chart
Rc: Red cell
Ch: Chlorophyll
An: Anthocyanin
Ca: Carotenoid
PS I: Photosystem I
PS II: Photosystem II
LHC: The light harvesting complex
DPMs: Differentially produced metabolites
DEGs: Differentially expressed genes
PCC: Pearson correlation coefficient
DHQ: Dihydroquercetin
DHM: Dihydromyricetin
DHK: Dihydrokaempferol
