# Supplementary material for: Metabolome and transcriptome profiling reveals anthocyanin contents and anthocyanin-related genes of chimeric leaves in Ananas comosus var. bracteatus
Source: BMC Genomics. 2021 May 7;22:331. doi: 10.1186/s12864-021-07642-x (PMC8105979; doi:10.1186/s12864-021-07642-x)
Supplement: Supplementary file 1 — Additional file 1: File S1: Annotated KEGG maps of metabolites. Blue plots indicate no significant changes between GR and RE samples. Red/green plots indicate up/down production of metabolites in RE samples compared with GR samples. White plots indicated undetectable metabolites. [file 12864_2021_7642_MOESM1_ESM.zip › File S1/ko00970.html]

KEGG PATHWAY: Pathway ko00970
