# Supplementary figures and images for: Metabolome and transcriptome profiling reveals anthocyanin contents and anthocyanin-related genes of chimeric leaves in Ananas comosus var. bracteatus
Source: BMC Genomics. 2021 May 7;22:331. doi: 10.1186/s12864-021-07642-x (PMC8105979; doi:10.1186/s12864-021-07642-x)

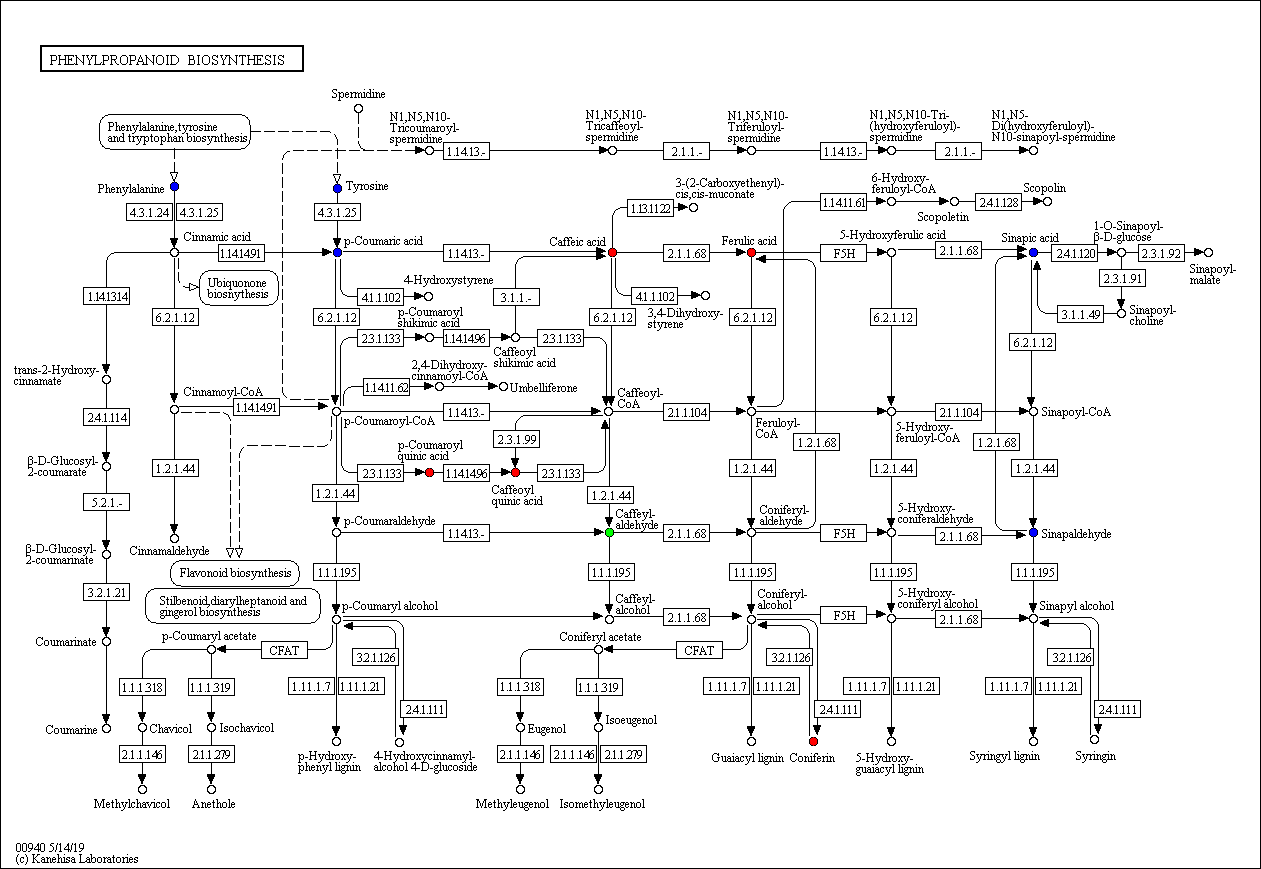

Supplement: Supplementary file 1 — Additional file 1: File S1: Annotated KEGG maps of metabolites. Blue plots indicate no significant changes between GR and RE samples. Red/green plots indicate up/down production of metabolites in RE samples compared with GR samples. White plots indicated undetectable metabolites. [file 12864_2021_7642_MOESM1_ESM.zip › File S1/ko00940.png]

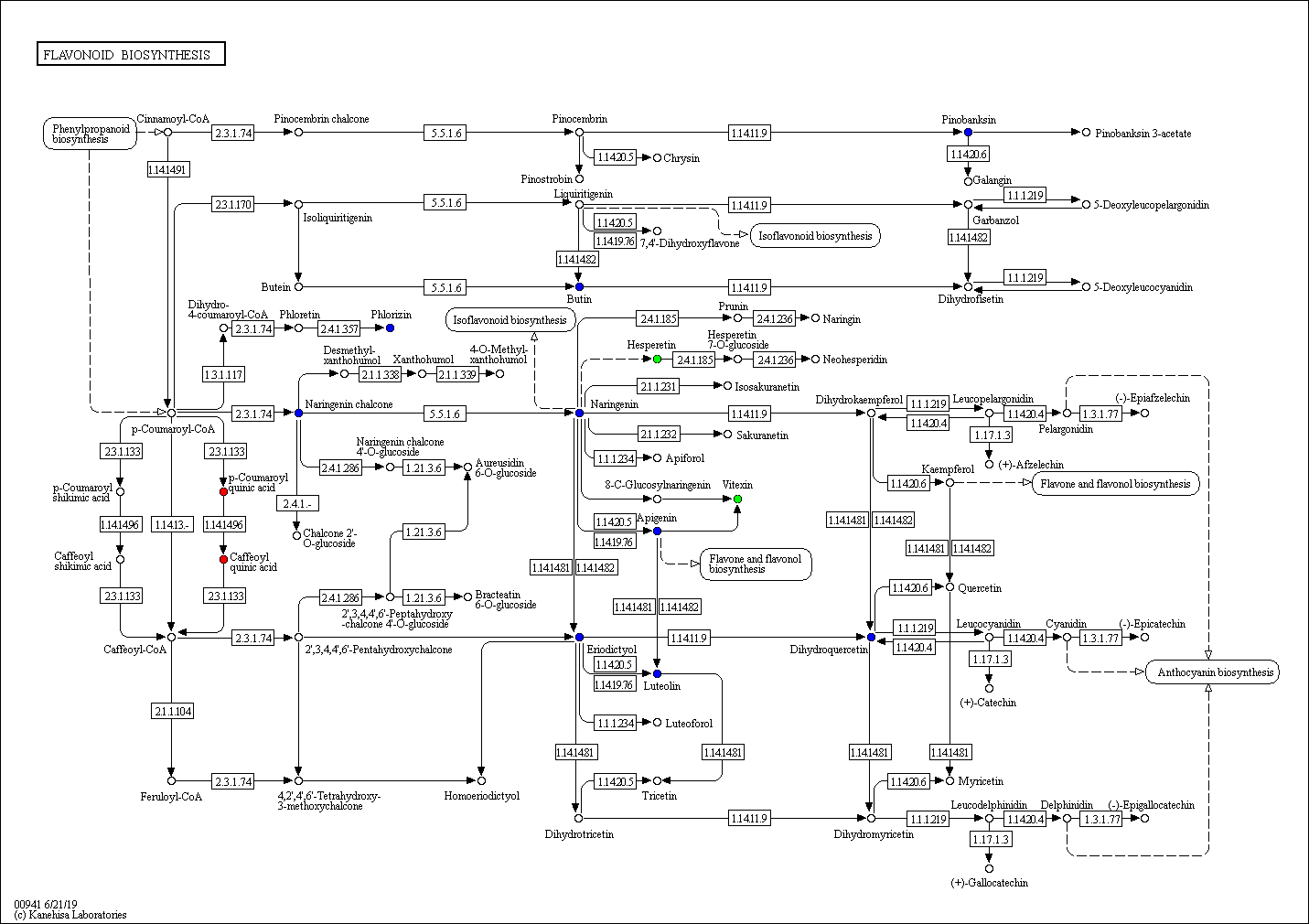

Supplement: Supplementary file 1 — Additional file 1: File S1: Annotated KEGG maps of metabolites. Blue plots indicate no significant changes between GR and RE samples. Red/green plots indicate up/down production of metabolites in RE samples compared with GR samples. White plots indicated undetectable metabolites. [file 12864_2021_7642_MOESM1_ESM.zip › File S1/ko00941.png]

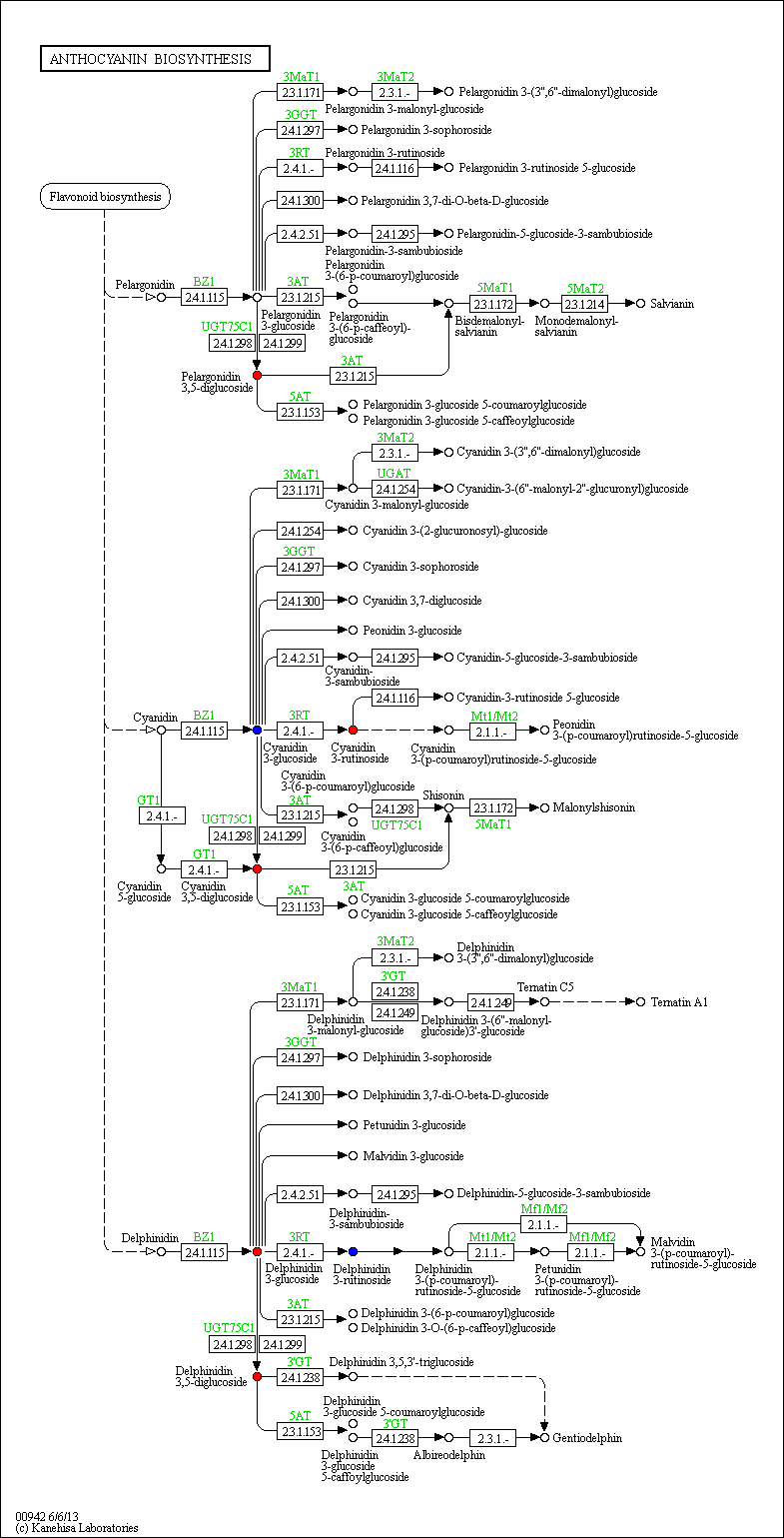

Supplement: Supplementary file 1 — Additional file 1: File S1: Annotated KEGG maps of metabolites. Blue plots indicate no significant changes between GR and RE samples. Red/green plots indicate up/down production of metabolites in RE samples compared with GR samples. White plots indicated undetectable metabolites. [file 12864_2021_7642_MOESM1_ESM.zip › File S1/ko00942.png]

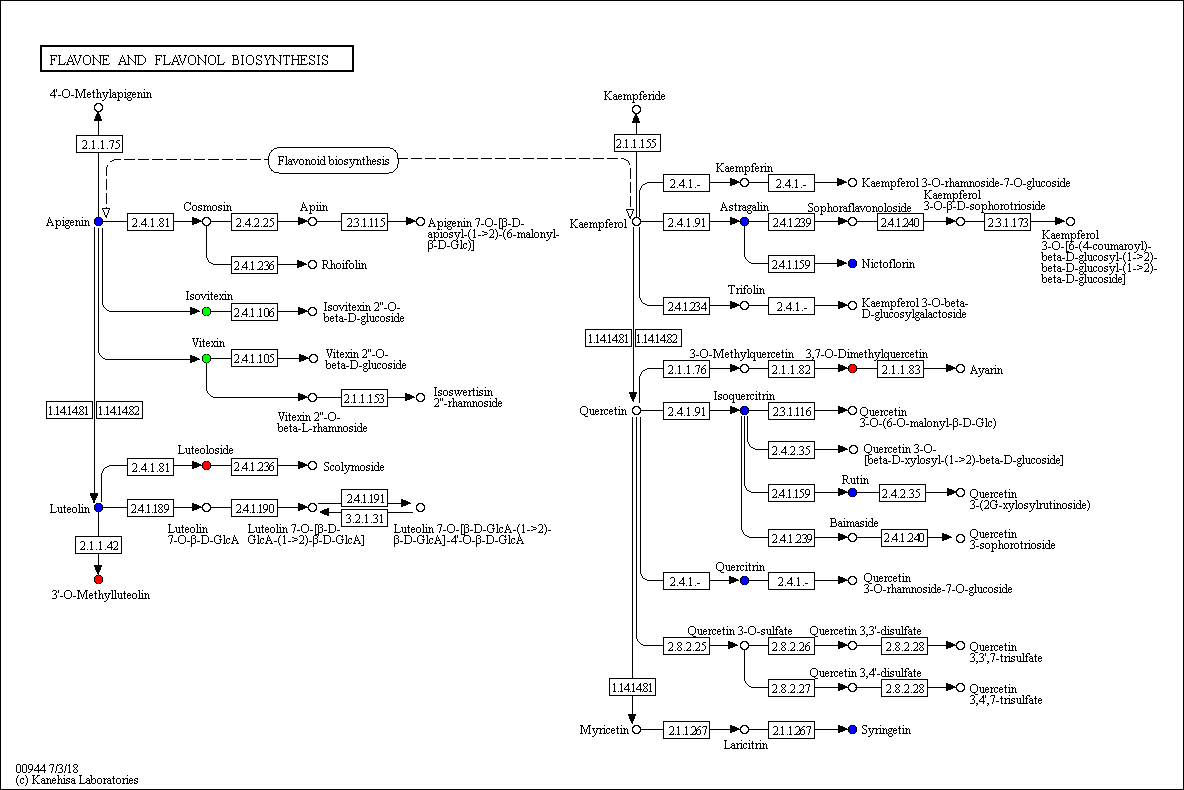

Supplement: Supplementary file 1 — Additional file 1: File S1: Annotated KEGG maps of metabolites. Blue plots indicate no significant changes between GR and RE samples. Red/green plots indicate up/down production of metabolites in RE samples compared with GR samples. White plots indicated undetectable metabolites. [file 12864_2021_7642_MOESM1_ESM.zip › File S1/ko00944.png]

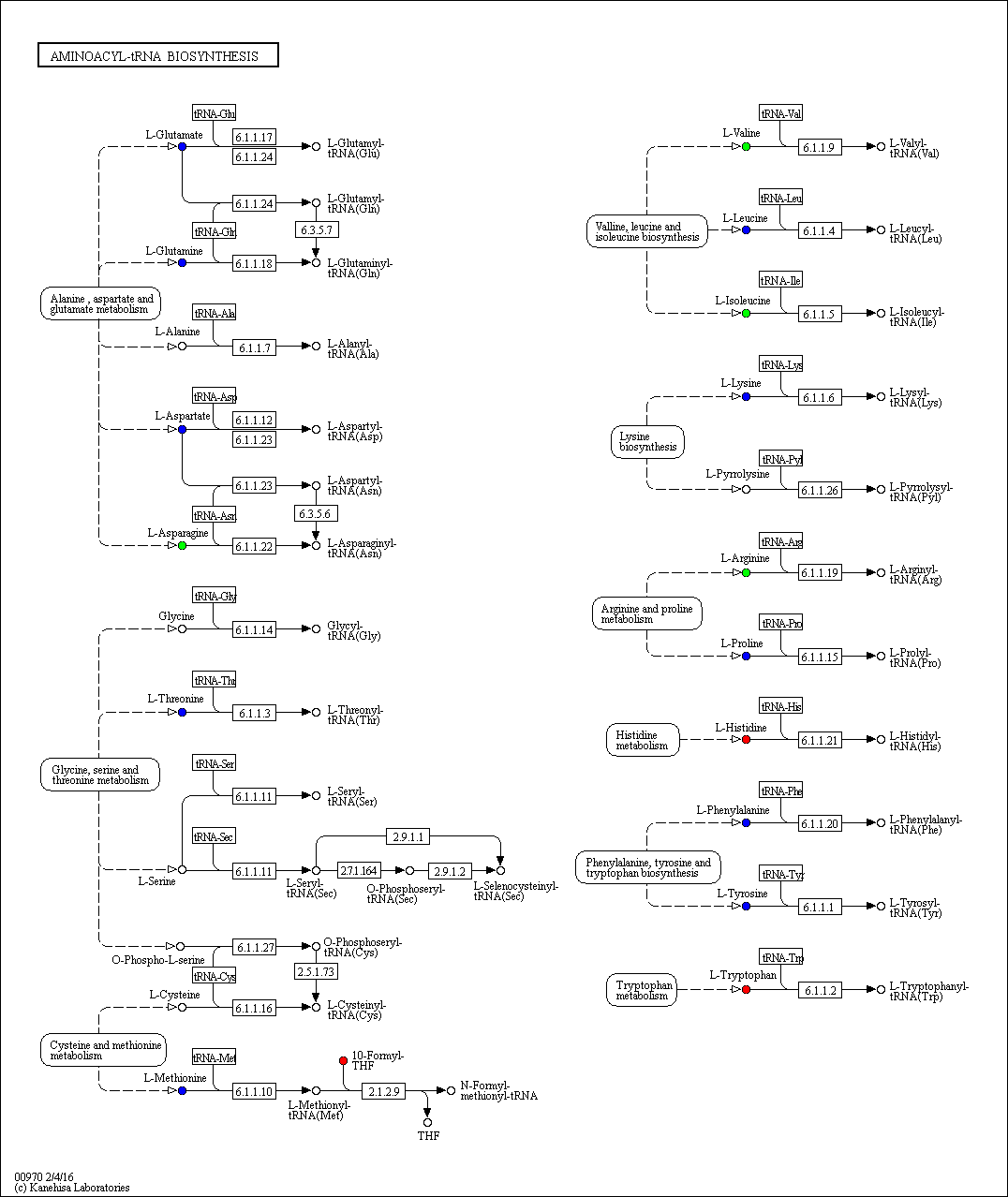

Supplement: Supplementary file 1 — Additional file 1: File S1: Annotated KEGG maps of metabolites. Blue plots indicate no significant changes between GR and RE samples. Red/green plots indicate up/down production of metabolites in RE samples compared with GR samples. White plots indicated undetectable metabolites. [file 12864_2021_7642_MOESM1_ESM.zip › File S1/ko00970.png]

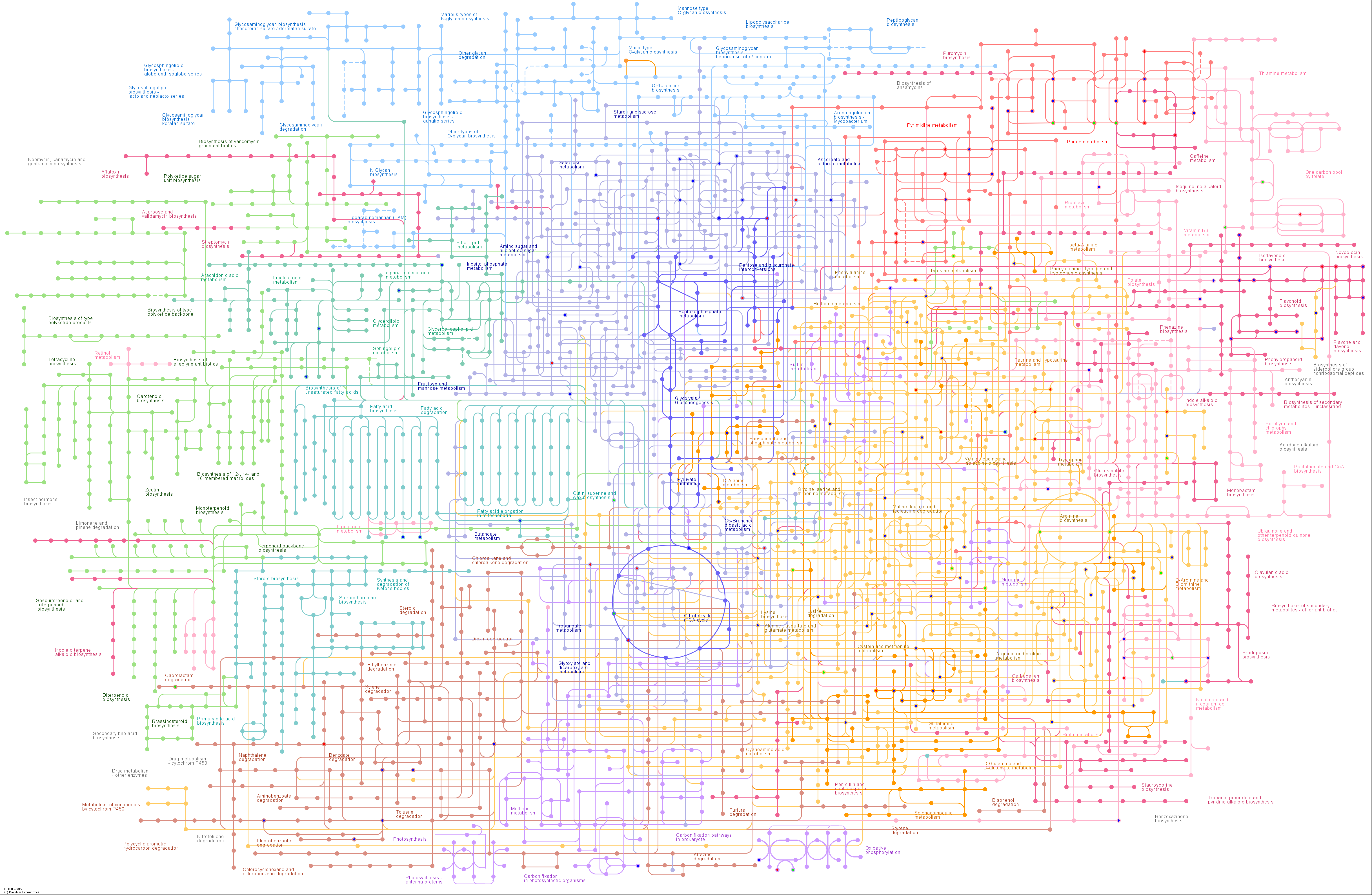

Supplement: Supplementary file 1 — Additional file 1: File S1: Annotated KEGG maps of metabolites. Blue plots indicate no significant changes between GR and RE samples. Red/green plots indicate up/down production of metabolites in RE samples compared with GR samples. White plots indicated undetectable metabolites. [file 12864_2021_7642_MOESM1_ESM.zip › File S1/ko01100.png]

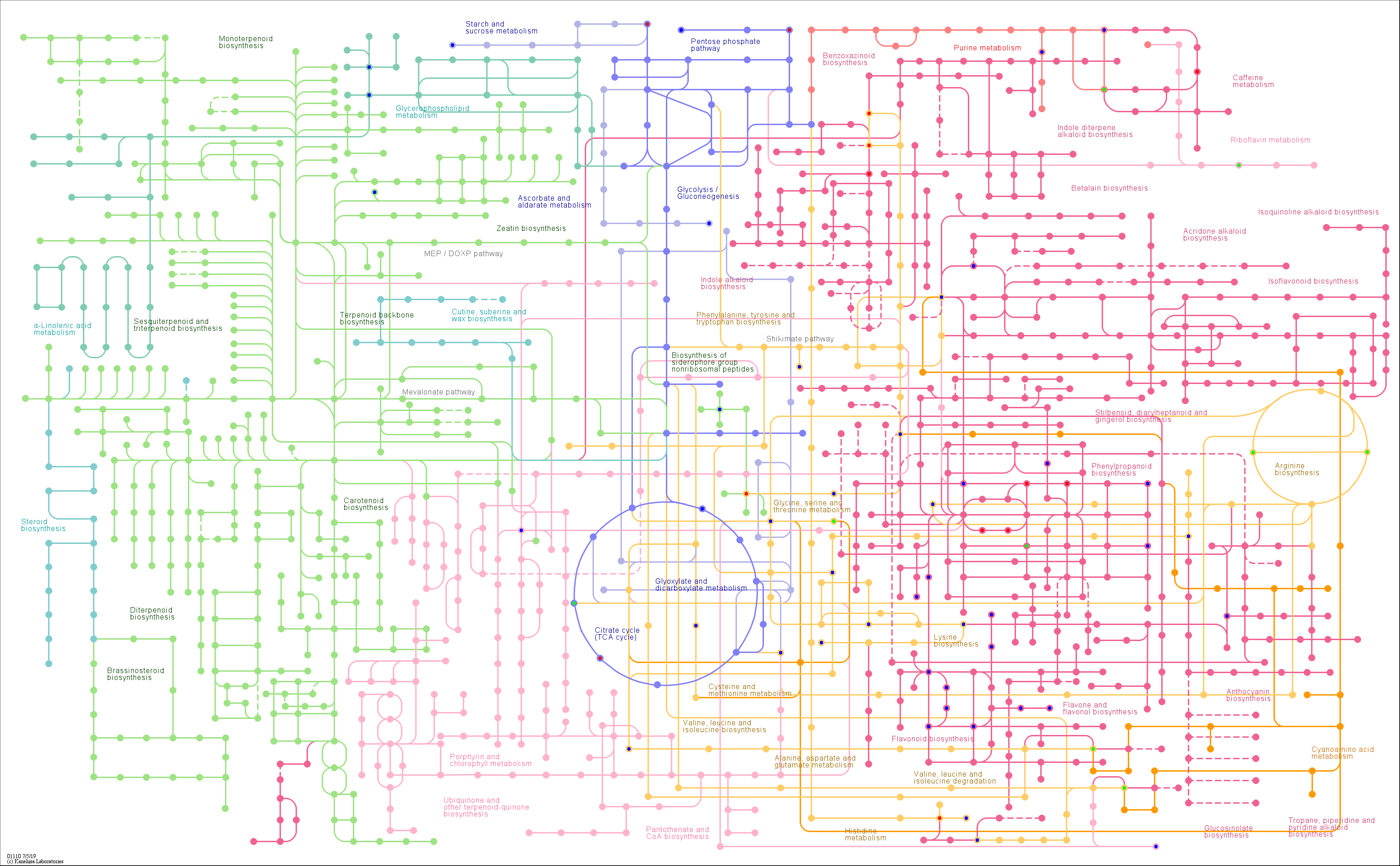

Supplement: Supplementary file 1 — Additional file 1: File S1: Annotated KEGG maps of metabolites. Blue plots indicate no significant changes between GR and RE samples. Red/green plots indicate up/down production of metabolites in RE samples compared with GR samples. White plots indicated undetectable metabolites. [file 12864_2021_7642_MOESM1_ESM.zip › File S1/ko01110.png]

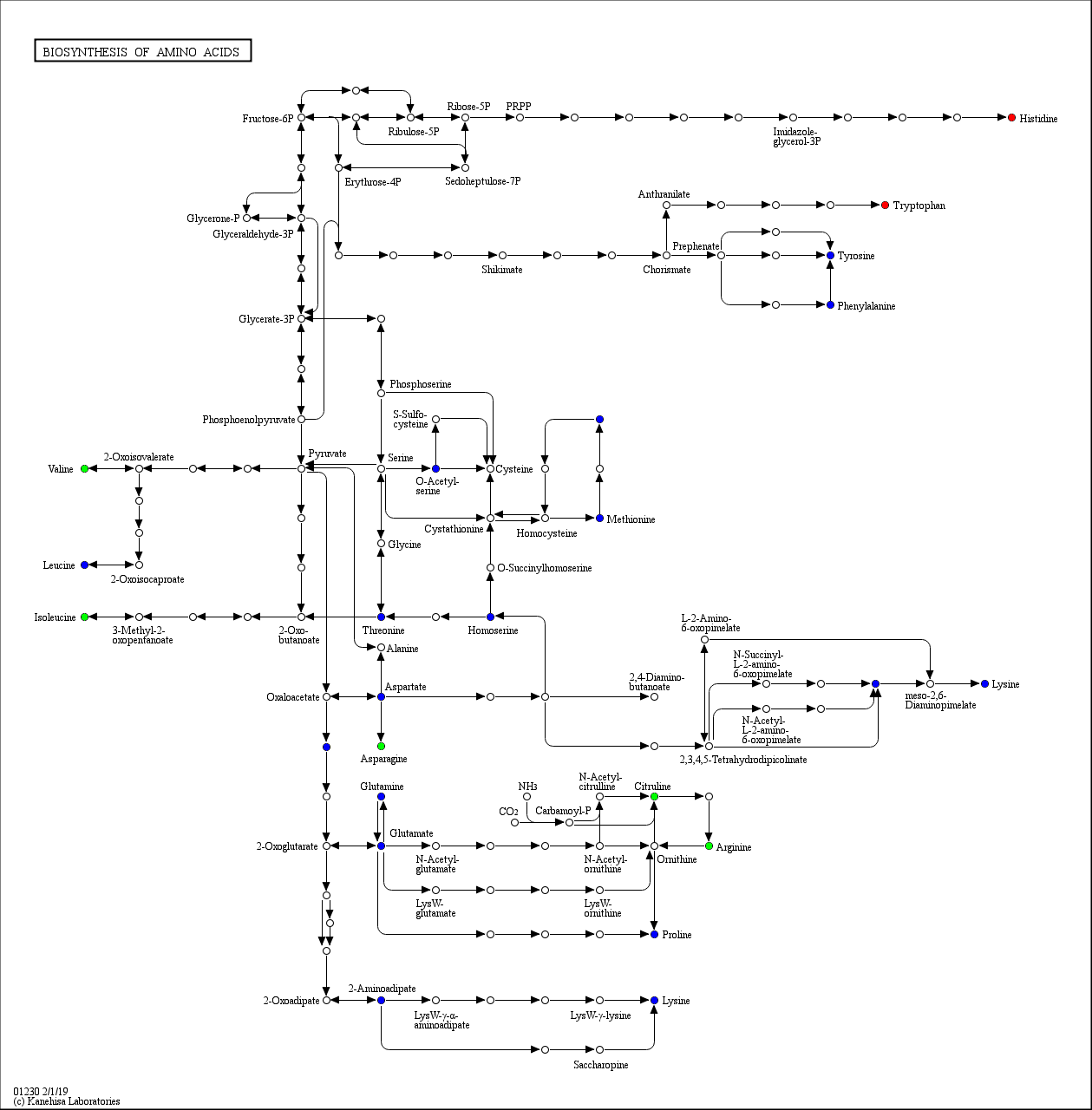

Supplement: Supplementary file 1 — Additional file 1: File S1: Annotated KEGG maps of metabolites. Blue plots indicate no significant changes between GR and RE samples. Red/green plots indicate up/down production of metabolites in RE samples compared with GR samples. White plots indicated undetectable metabolites. [file 12864_2021_7642_MOESM1_ESM.zip › File S1/ko01230.png]

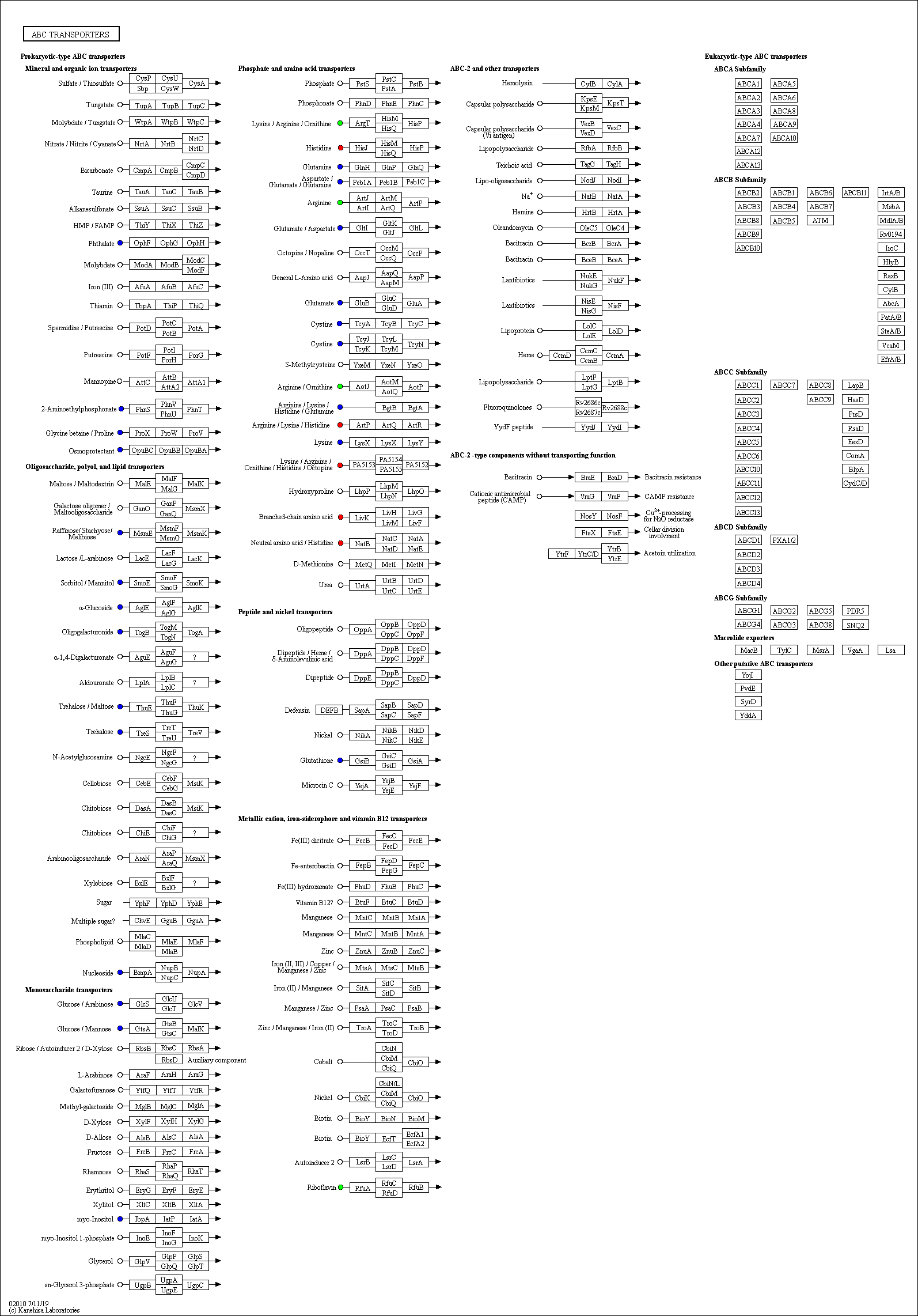

Supplement: Supplementary file 1 — Additional file 1: File S1: Annotated KEGG maps of metabolites. Blue plots indicate no significant changes between GR and RE samples. Red/green plots indicate up/down production of metabolites in RE samples compared with GR samples. White plots indicated undetectable metabolites. [file 12864_2021_7642_MOESM1_ESM.zip › File S1/ko02010.png]

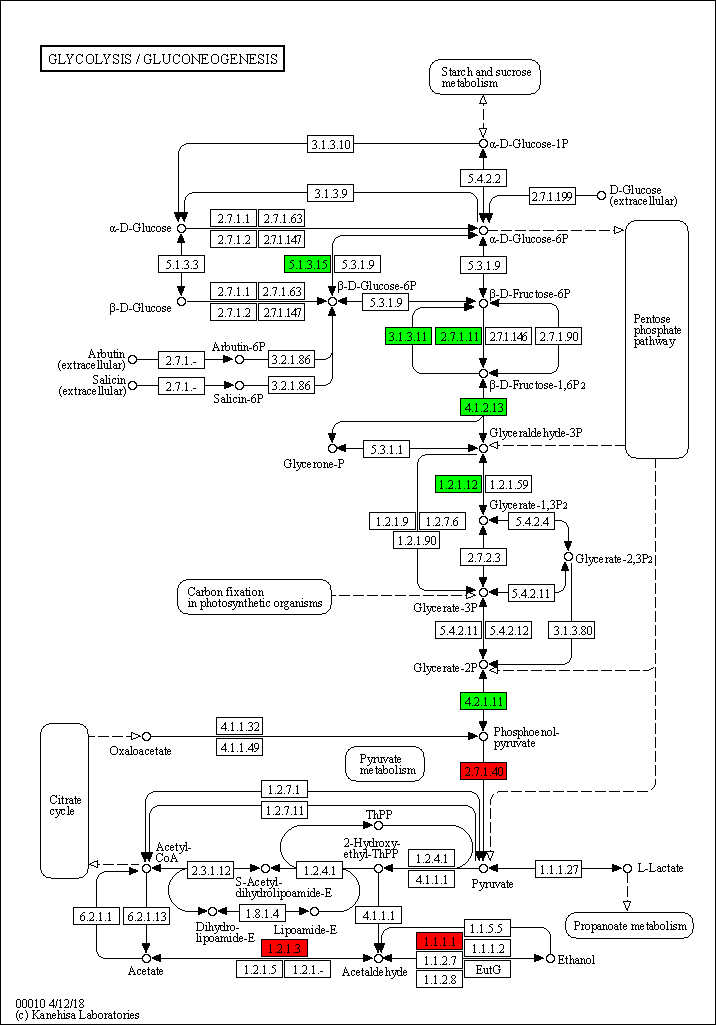

Supplement: Supplementary file 2 — Additional file 2: File S2: Annotated KEGG maps of genes. Blue bars indicate no significant changes between GR and RE samples. Red/green bars indicate up/down regulation of genes in RE samples compared with GR samples. White bars indicated undetectable genes. [file 12864_2021_7642_MOESM2_ESM.zip › File S2/ko00010.png]

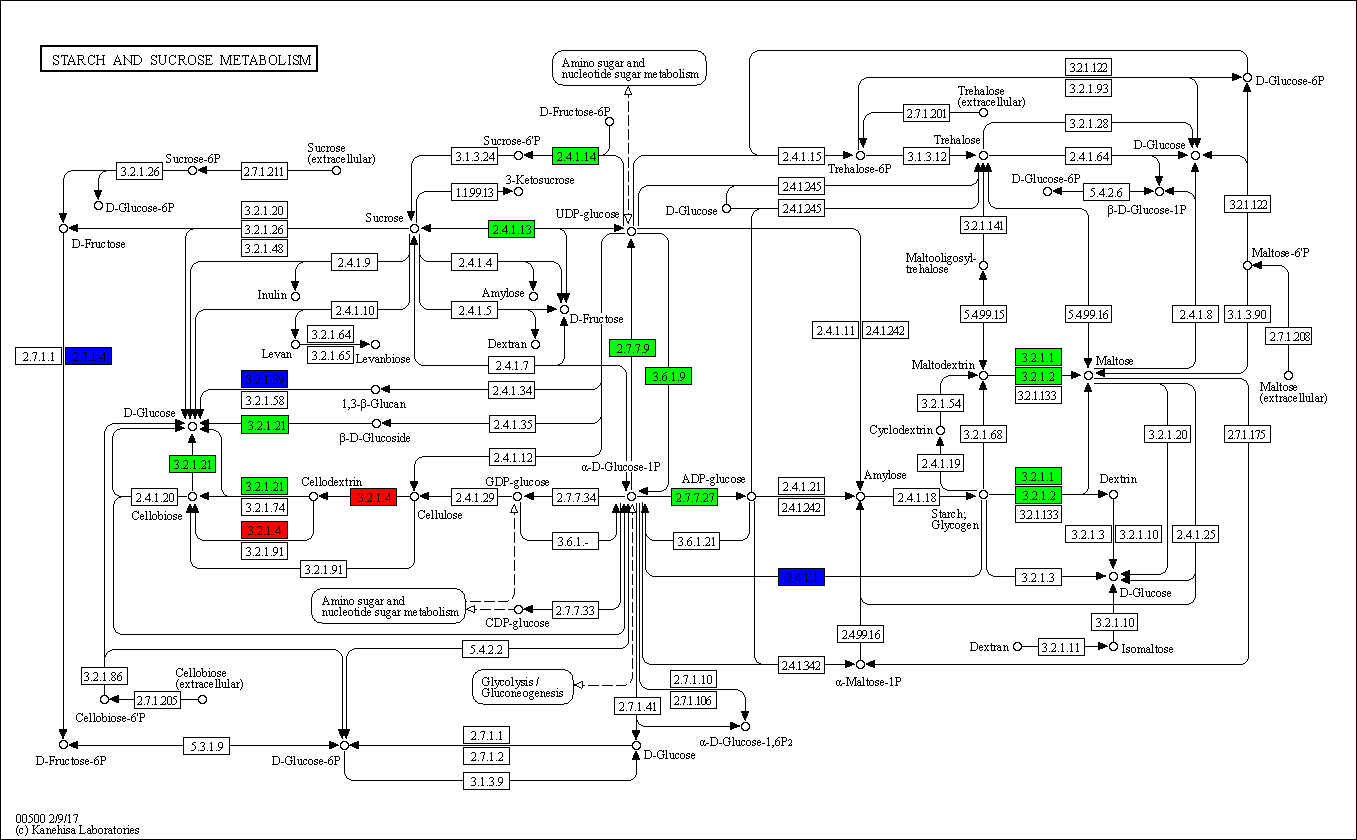

Supplement: Supplementary file 2 — Additional file 2: File S2: Annotated KEGG maps of genes. Blue bars indicate no significant changes between GR and RE samples. Red/green bars indicate up/down regulation of genes in RE samples compared with GR samples. White bars indicated undetectable genes. [file 12864_2021_7642_MOESM2_ESM.zip › File S2/ko00500.png]

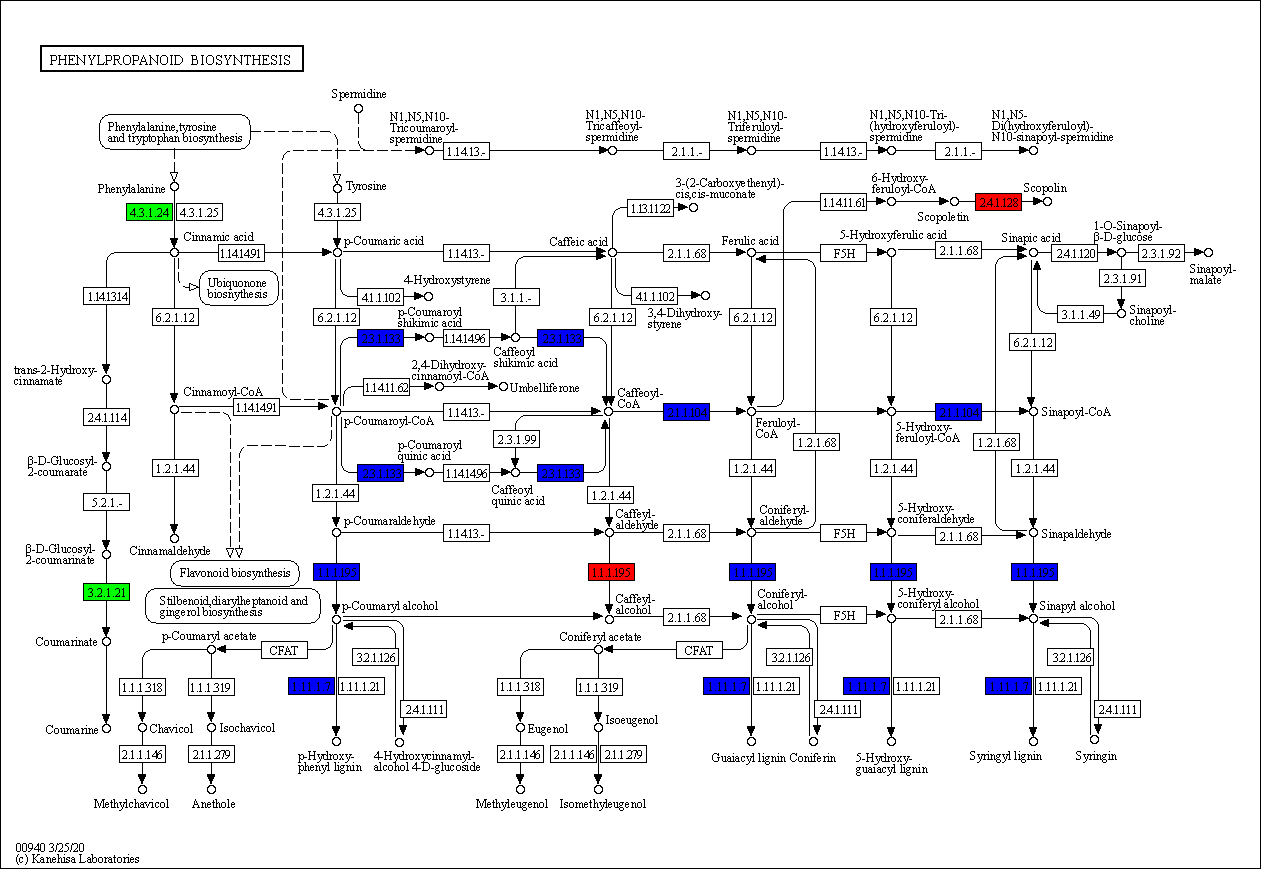

Supplement: Supplementary file 2 — Additional file 2: File S2: Annotated KEGG maps of genes. Blue bars indicate no significant changes between GR and RE samples. Red/green bars indicate up/down regulation of genes in RE samples compared with GR samples. White bars indicated undetectable genes. [file 12864_2021_7642_MOESM2_ESM.zip › File S2/ko00940.png]

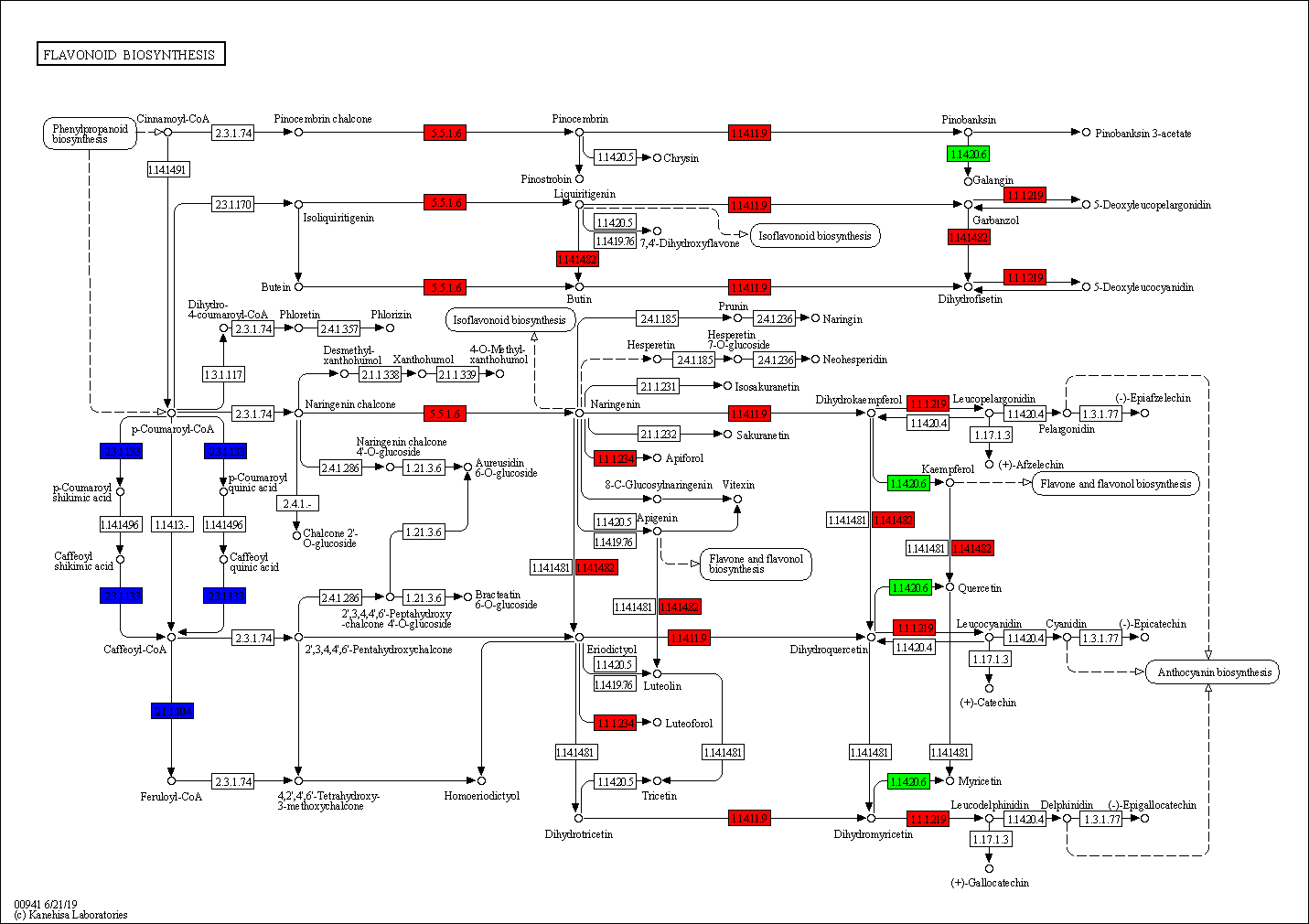

Supplement: Supplementary file 2 — Additional file 2: File S2: Annotated KEGG maps of genes. Blue bars indicate no significant changes between GR and RE samples. Red/green bars indicate up/down regulation of genes in RE samples compared with GR samples. White bars indicated undetectable genes. [file 12864_2021_7642_MOESM2_ESM.zip › File S2/ko00941.png]

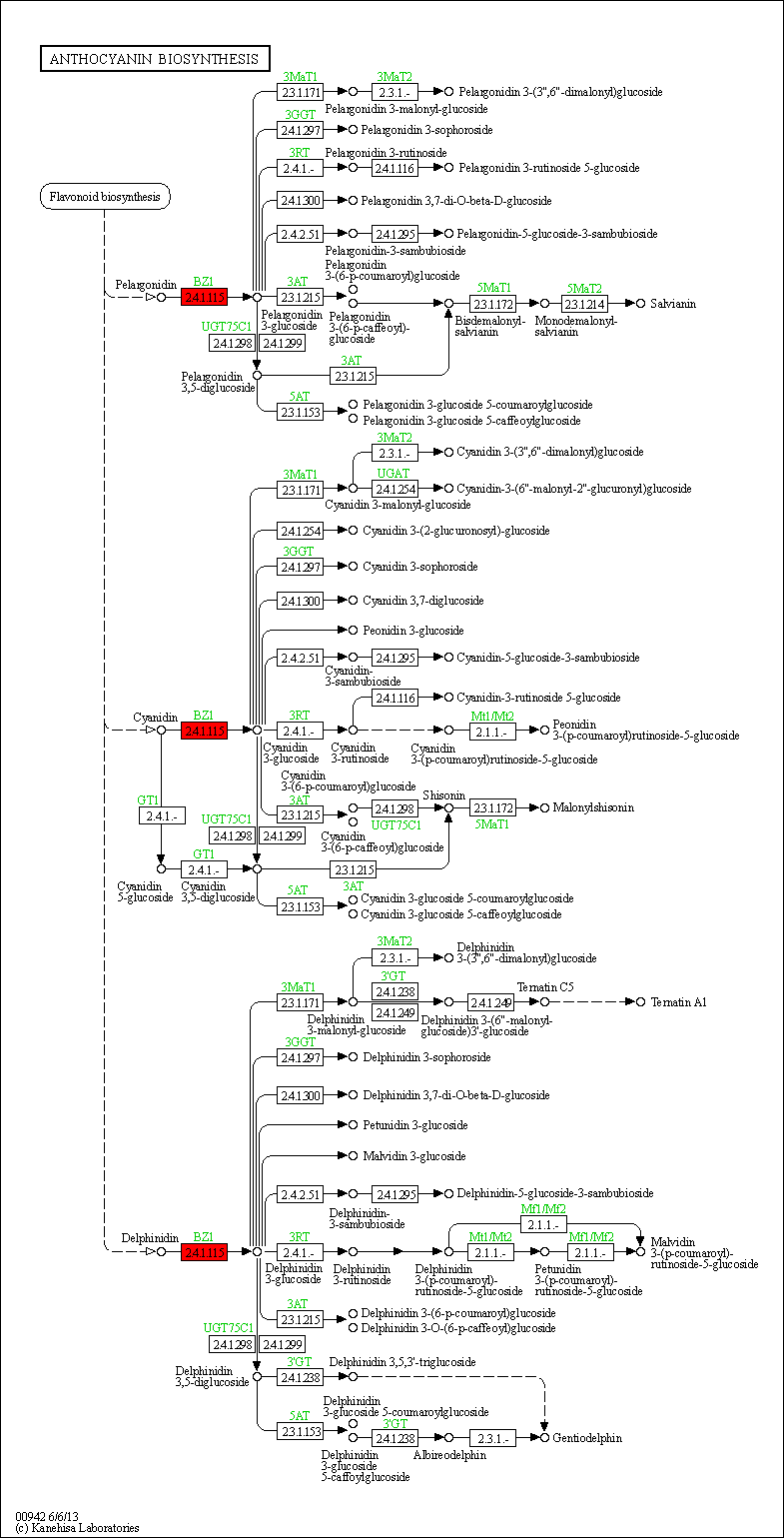

Supplement: Supplementary file 2 — Additional file 2: File S2: Annotated KEGG maps of genes. Blue bars indicate no significant changes between GR and RE samples. Red/green bars indicate up/down regulation of genes in RE samples compared with GR samples. White bars indicated undetectable genes. [file 12864_2021_7642_MOESM2_ESM.zip › File S2/ko00942.png]

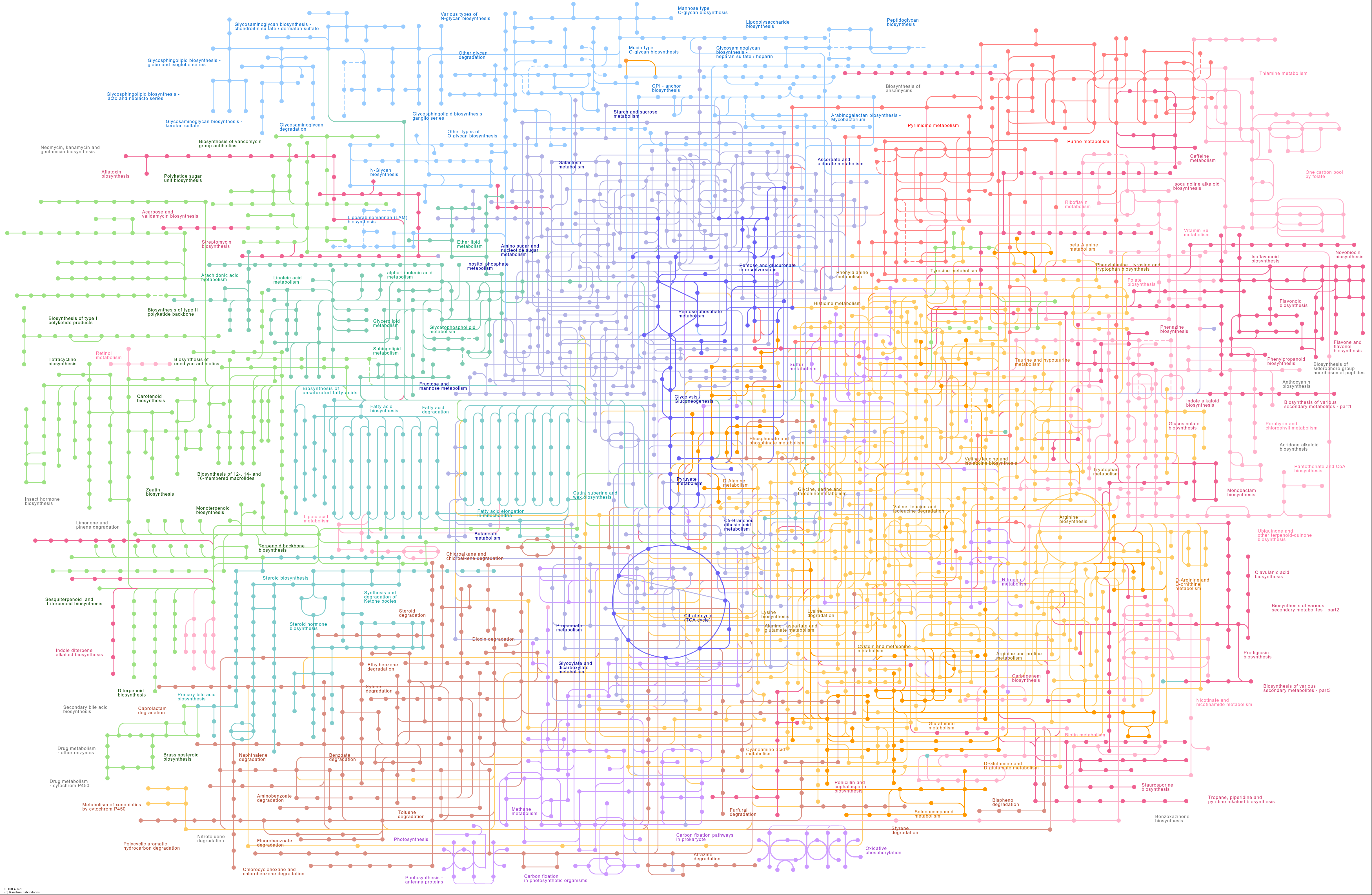

Supplement: Supplementary file 2 — Additional file 2: File S2: Annotated KEGG maps of genes. Blue bars indicate no significant changes between GR and RE samples. Red/green bars indicate up/down regulation of genes in RE samples compared with GR samples. White bars indicated undetectable genes. [file 12864_2021_7642_MOESM2_ESM.zip › File S2/ko01100.png]

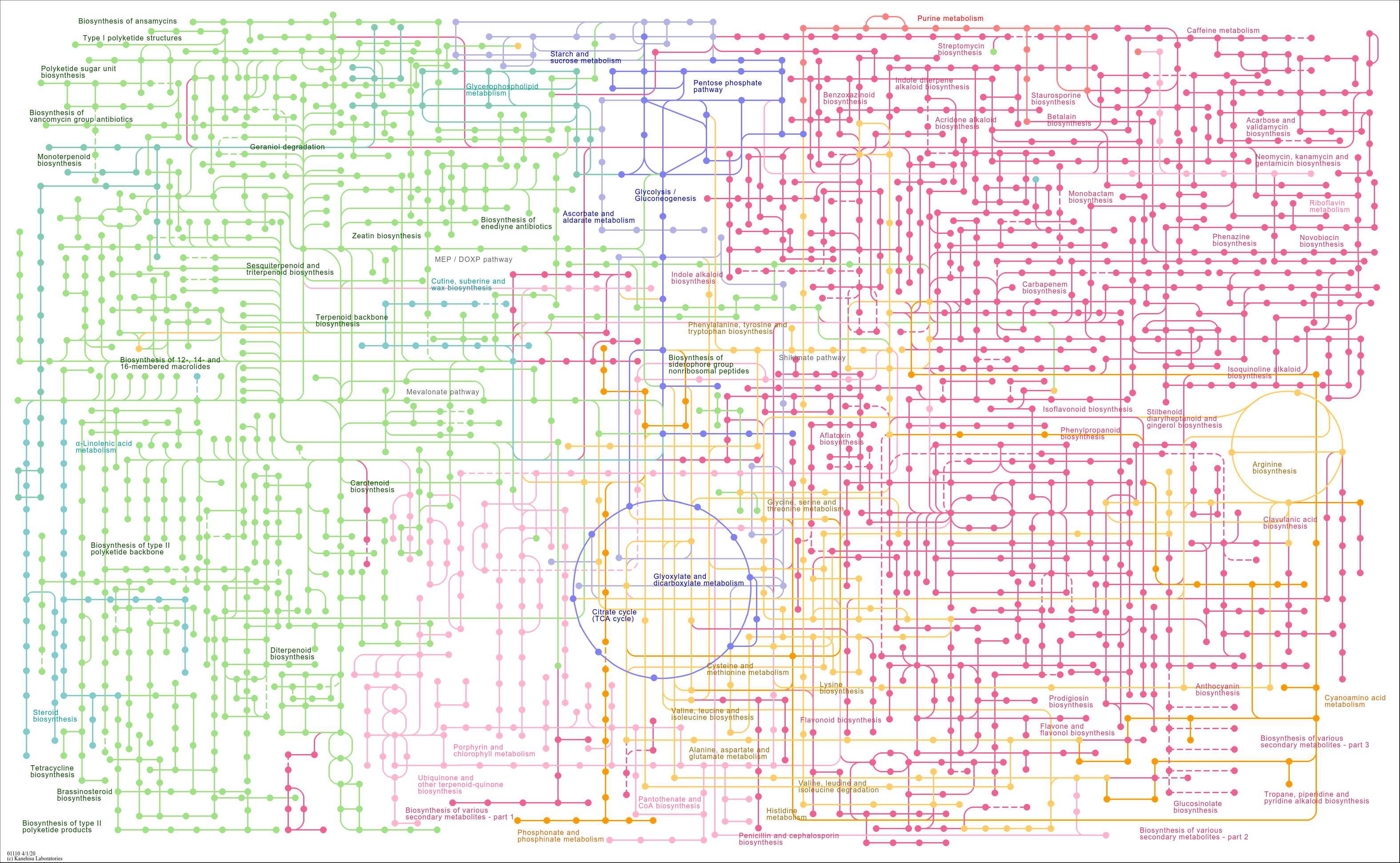

Supplement: Supplementary file 2 — Additional file 2: File S2: Annotated KEGG maps of genes. Blue bars indicate no significant changes between GR and RE samples. Red/green bars indicate up/down regulation of genes in RE samples compared with GR samples. White bars indicated undetectable genes. [file 12864_2021_7642_MOESM2_ESM.zip › File S2/ko01110.png]

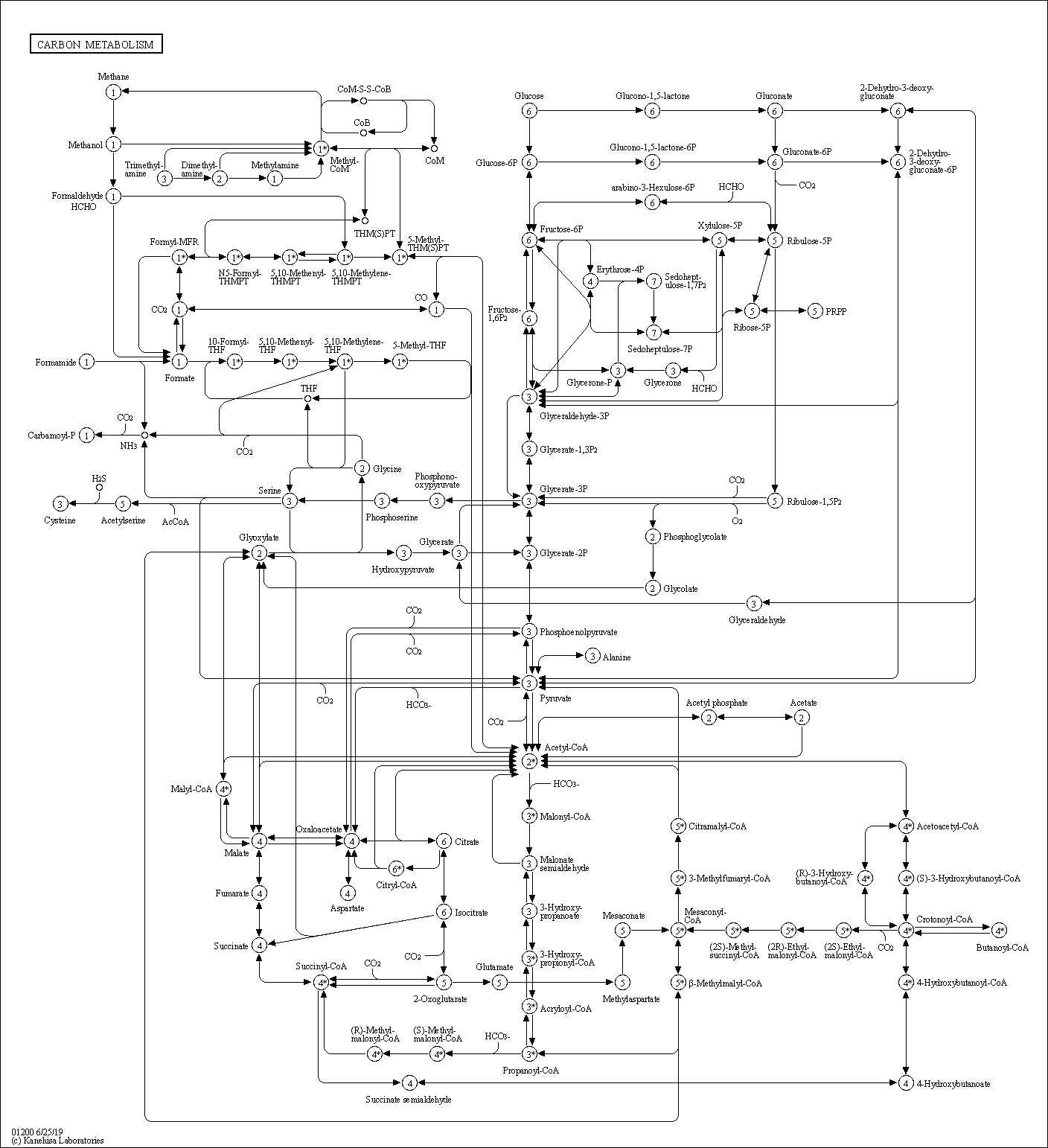

Supplement: Supplementary file 2 — Additional file 2: File S2: Annotated KEGG maps of genes. Blue bars indicate no significant changes between GR and RE samples. Red/green bars indicate up/down regulation of genes in RE samples compared with GR samples. White bars indicated undetectable genes. [file 12864_2021_7642_MOESM2_ESM.zip › File S2/ko01200.png]

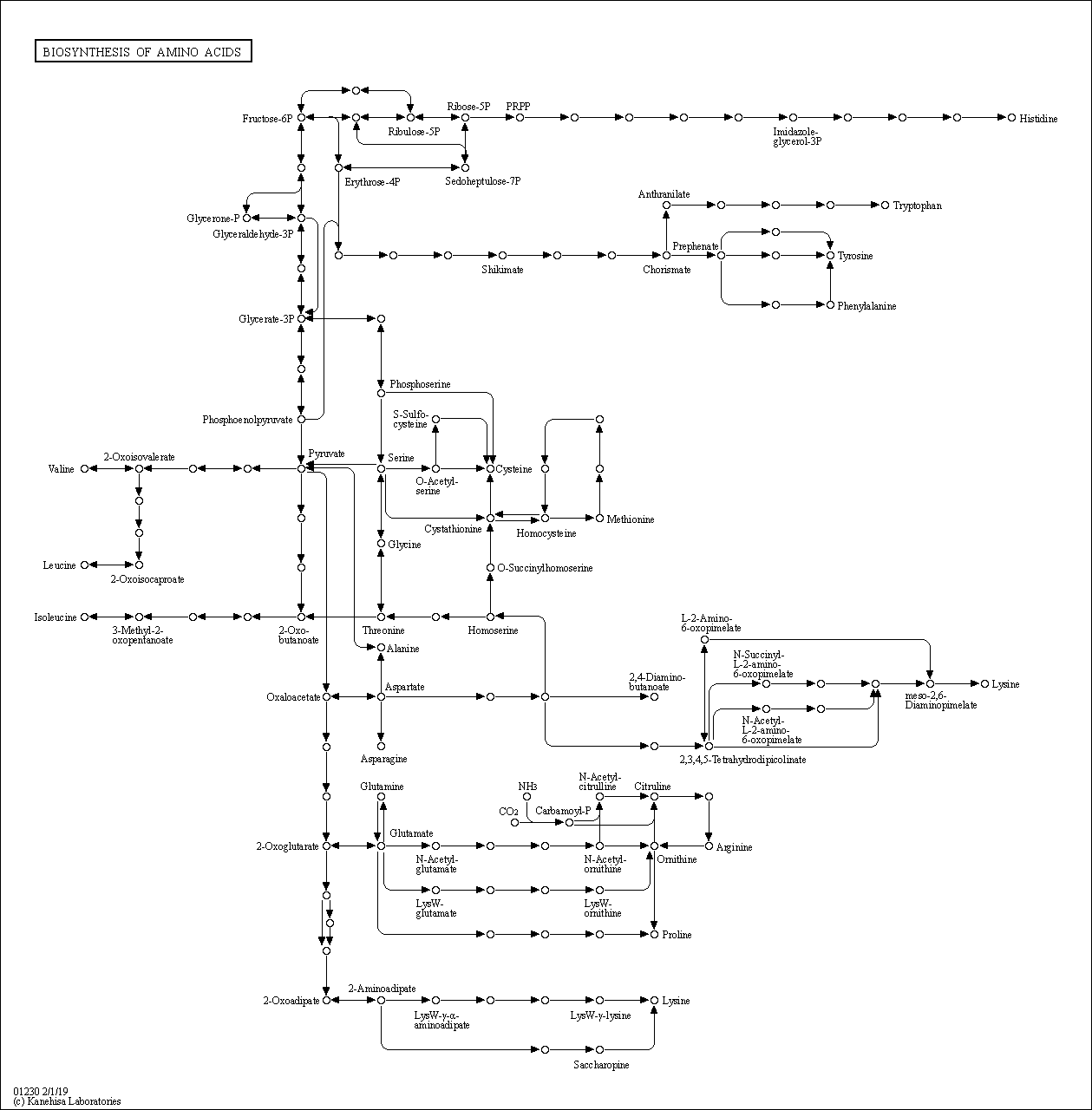

Supplement: Supplementary file 2 — Additional file 2: File S2: Annotated KEGG maps of genes. Blue bars indicate no significant changes between GR and RE samples. Red/green bars indicate up/down regulation of genes in RE samples compared with GR samples. White bars indicated undetectable genes. [file 12864_2021_7642_MOESM2_ESM.zip › File S2/ko01230.png]

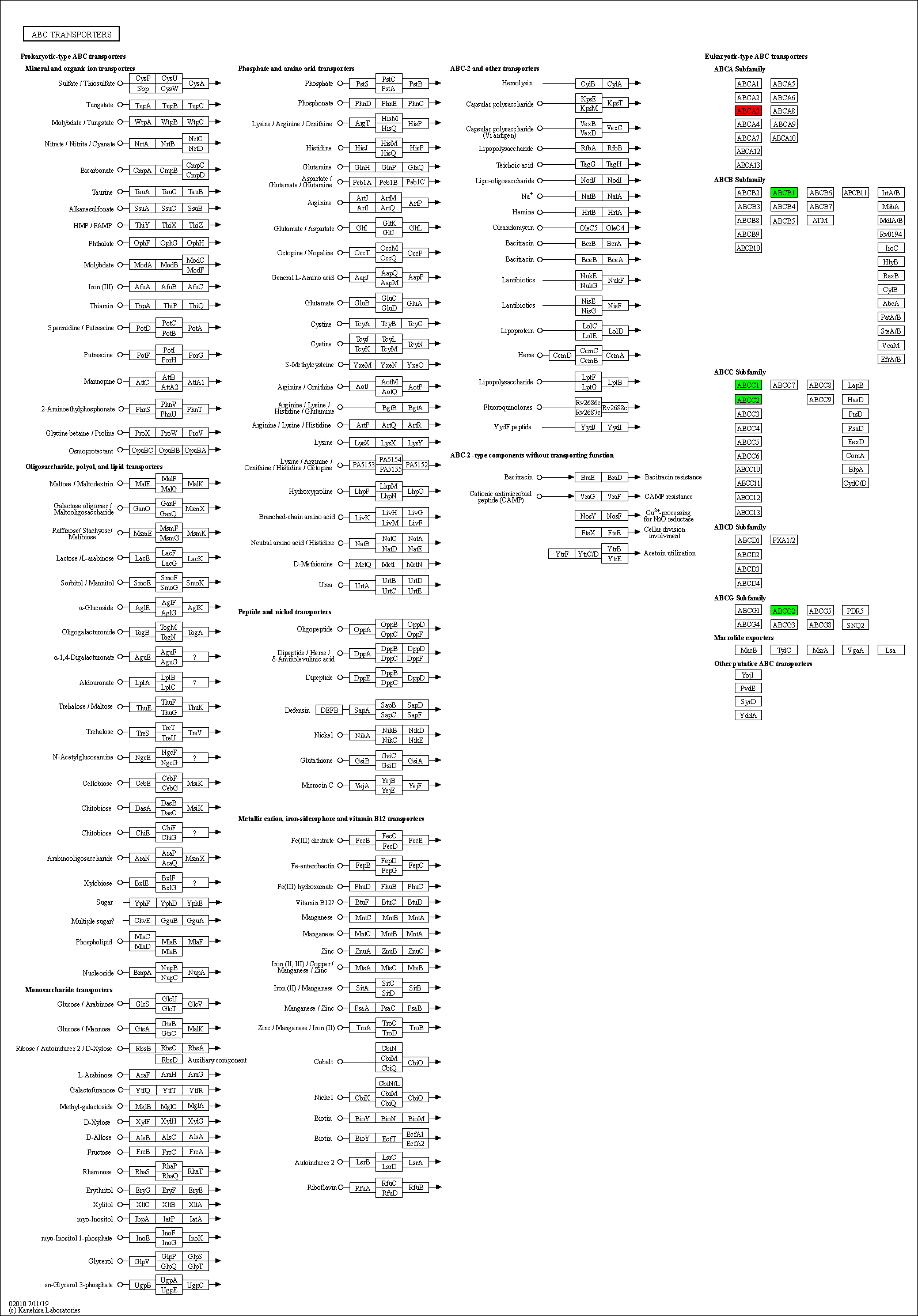

Supplement: Supplementary file 2 — Additional file 2: File S2: Annotated KEGG maps of genes. Blue bars indicate no significant changes between GR and RE samples. Red/green bars indicate up/down regulation of genes in RE samples compared with GR samples. White bars indicated undetectable genes. [file 12864_2021_7642_MOESM2_ESM.zip › File S2/ko02010.png]

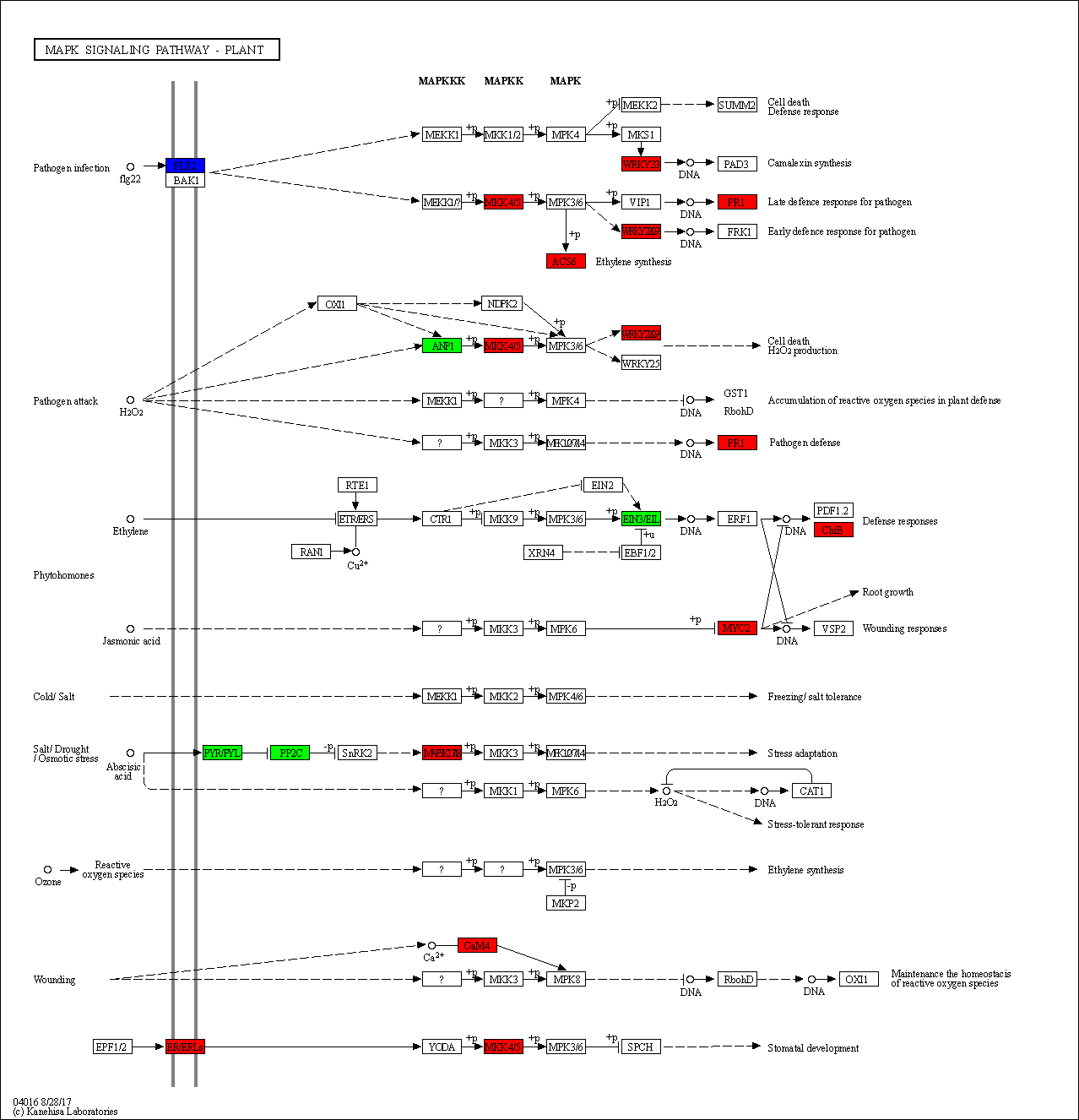

Supplement: Supplementary file 2 — Additional file 2: File S2: Annotated KEGG maps of genes. Blue bars indicate no significant changes between GR and RE samples. Red/green bars indicate up/down regulation of genes in RE samples compared with GR samples. White bars indicated undetectable genes. [file 12864_2021_7642_MOESM2_ESM.zip › File S2/ko04016.png]

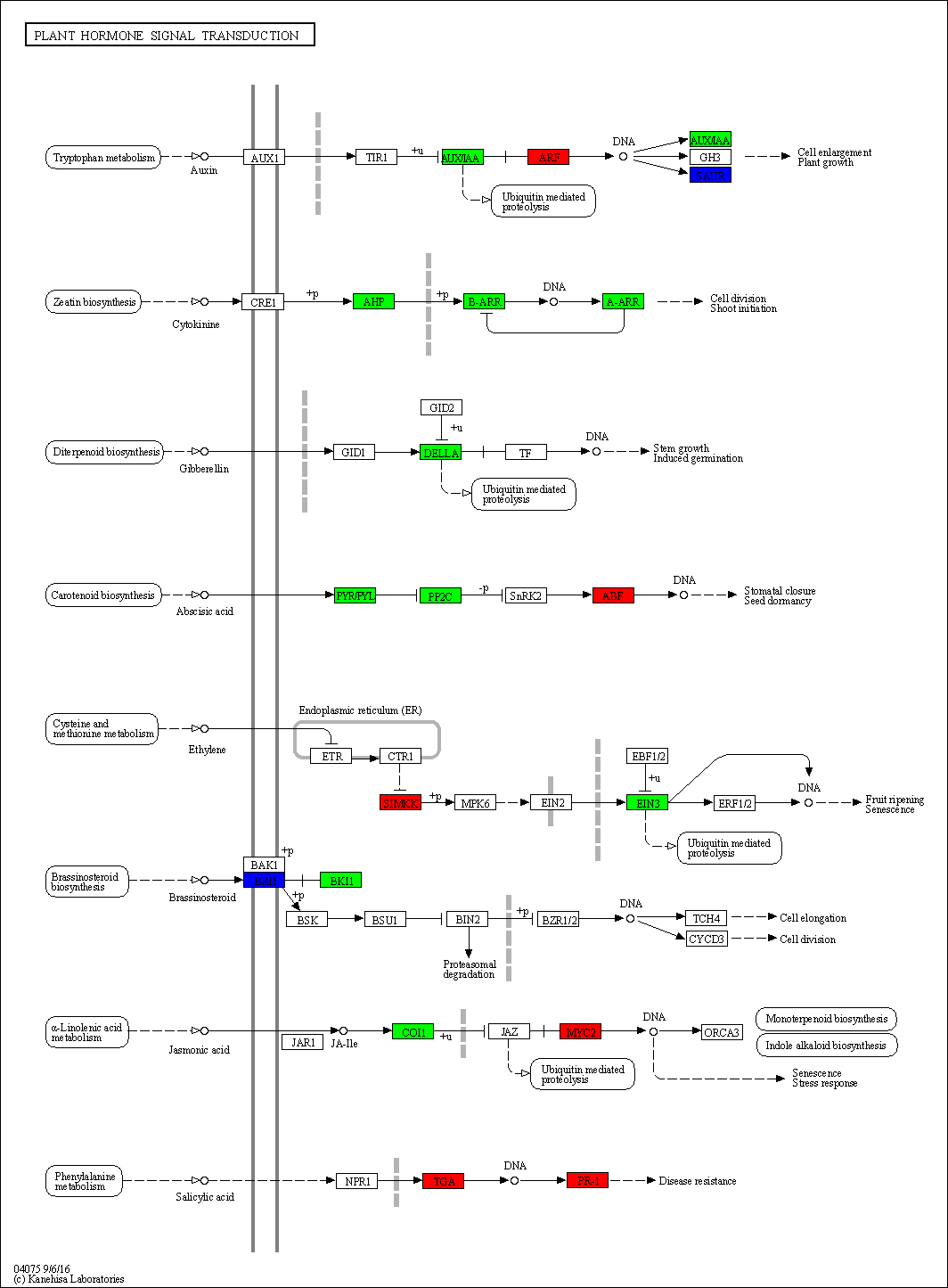

Supplement: Supplementary file 2 — Additional file 2: File S2: Annotated KEGG maps of genes. Blue bars indicate no significant changes between GR and RE samples. Red/green bars indicate up/down regulation of genes in RE samples compared with GR samples. White bars indicated undetectable genes. [file 12864_2021_7642_MOESM2_ESM.zip › File S2/ko04075.png]

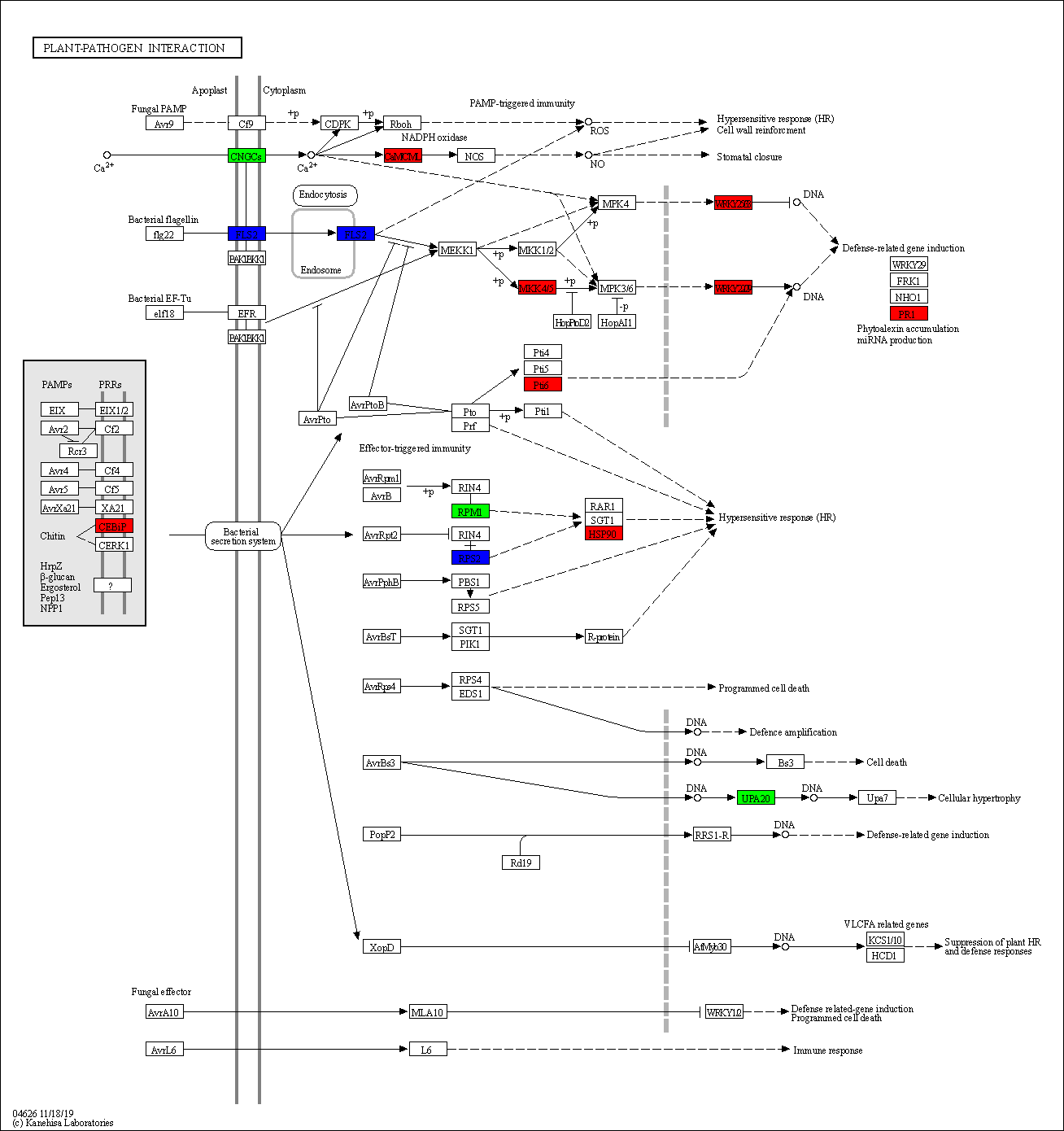

Supplement: Supplementary file 2 — Additional file 2: File S2: Annotated KEGG maps of genes. Blue bars indicate no significant changes between GR and RE samples. Red/green bars indicate up/down regulation of genes in RE samples compared with GR samples. White bars indicated undetectable genes. [file 12864_2021_7642_MOESM2_ESM.zip › File S2/ko04626.png]
