# Supplementary material for: Metabolome and transcriptome profiling reveals anthocyanin contents and anthocyanin-related genes of chimeric leaves in Ananas comosus var. bracteatus
Source: BMC Genomics. 2021 May 7;22:331. doi: 10.1186/s12864-021-07642-x (PMC8105979; doi:10.1186/s12864-021-07642-x)
Supplement: Supplementary file 2 — Additional file 2: File S2: Annotated KEGG maps of genes. Blue bars indicate no significant changes between GR and RE samples. Red/green bars indicate up/down regulation of genes in RE samples compared with GR samples. White bars indicated undetectable genes. [file 12864_2021_7642_MOESM2_ESM.zip › File S2/ko00941.html]

ko00941
